# Perceptions of food environments in the school and at home during Covid-19: An online cross-sectional study of parents, teachers and experts from Latin America

**DOI:** 10.1371/journal.pone.0287747

**Published:** 2023-06-29

**Authors:** Marcos Galván, Jhazmín Hernández-Cabrera, Guadalupe López-Rodríguez, Nelly Bustos, Rubén García-Cruz, Rebeca Guzmán-Saldaña, Teresita Alzate-Yepes, Oscar Galván-Valencia

**Affiliations:** 1 Interdisciplinary Health Sciences Research Center, Universidad Autónoma del Estado de Hidalgo, Pachuca, Mexico; 2 Interdisciplinary Network of Experts in School Environments in Latin America, Universidad Autónoma del Estado de Hidalgo, Pachuca, Mexico; 3 Nutrition and Food Technology Institute, University of Chile, Santiago, Chile; 4 School of Nutrition and Dietetics, University of Antioquia, Medellín, Colombia; 5 Center for Nutrition and Health Research, National Institute of Public Health, Cuernavaca, Mexico; Zagazig University Faculty of Human Medicine, EGYPT

## Abstract

**Background:**

The high prevalence of overweight and obesity in children from Latin America (LA) have been related to obesogenic food environments. Besides, the negative effects of the Covid-19 pandemic should also be considered. The objective of this research was to describe and compare the perceptions of parents, teachers, and experts in LA of food environments at home and school that favor healthy habits in schoolchildren in pre Covid-19 stage and during the pandemic.

**Methods:**

This study used a survey self-reporting regarding conditions at home and the school favoring healthy habits, for three profiles: parents, primary school teachers, and experts. A fisher exact test was used to establish the difference between the response categories between countries and profiles. Logistic regression models were used to determine the probability of response in the levels of importance adjusted for sex and nationality.

**Results:**

Information from 954 questionnaires was reported: 48.4% experts, 32.0% teachers, and 19.6% parents. There were differences in the perception of food environments at school between profiles (p<0.001). In multivariate logistic regression models, experts and teachers were 20% more likely to give greater importance to elements of the food environment at school compared to parents (p<0.001*)*.

**Conclusions:**

Our findings showed that parents were less likely to perceive important elements of the school food environment compared to experts and teachers. Interventions are required to improve healthy eating environments that consider children’s interpersonal mediators.

## Introduction

Before the Covid-19 pandemic, early-age overweight and obesity (OW/OB) was one of the growing public health problems with the greatest global impact, particularly in low- and medium-income countries [1]. Among children and adolescents from 0 to 19 years old, it has been reported that OW/OB affects 25% in Latin America (LA) [2].

The OW/OB is a very complex socio-medical phenomenon, its current approach requires a construction that considers the socio-ecological framework, the general theory of systems and the paradigm of complexity, to explain, from a systemic and integrative epistemology, multilevel frameworks, and the interaction of the variables that make them up, this is to consider the environment as well as the actors that affect people’s realities [3]. That said, to halt the increase in childhood obesity it is important to assume that its multidimensional character can be explained by applying models based on learning theories that can identify the processes of individual, social and environmental interaction that can prevent OW/OB [4].

At an organizational and community level, studying food environments in schools has been seen as relevant, as an essential resource in the prevention and containment of the growing infantile OW/OB pandemic. Multiple elements make up these environments, including school policies and infrastructure, food and nutrition education in and outside of the curriculum [5], health literacy among teachers (TE) [6] and their perceptions and attitudes about the subject [7], the food offered and the oversight of this food, school food programs (SFP), holistic school programs that promote healthy lifestyles, and support from parents (PA) and the community [2,8–10]. The interpersonal family environment has been identified as the area that most directly influences the behaviors and lifestyles of school-age children [10]. PA perceptions of their children’s eating habits are preponderant, and they may underestimate risky eating behaviors inside and outside the home [11].

It should be noted that during the Covid-19 pandemic, conditions for poor nutrition increased, both due to deficiency and excess, primarily in low- and medium-income countries [12]. Research in LA countries has reported that during the pandemic, children, adolescents and young adults increased their intake of high-energy dense foods such as candy and chips. Limited time outdoors led to a decrease in their physical activity (PhA) and an increase in sedentary activities such as screen use. These have been associated with an increase in body weight [13–16], as well as with changes in the behavior, sleep and emotional wellbeing of children and adolescents [17]. The Covid-19 pandemic left over 160 million students in LA and the Caribbean without in-person classes [18]. This not only interrupted their educational trajectories, but also negatively impacted their PhA, recreation, food and nutrition habits, since they also received PhA and food from SFP at school [19]. School closures forced homes to become schoolrooms. In poorer households, continuing the educational process at home was a challenge. The digital divide was evidenced by a lack of means for distancing learning, including adequate computers, internet connection, and the presence and supervision of a tutor [20].

This experience—of confinement, of homes converted into an extension of schools and educational life, and reports of behaviors harmful to students’ nutritional health—brought several needs to the front burner. These included the identification of methods and resources that can facilitate connections between the school and the home on various levels; the effort of the actors involved to strengthen the school as a fundamental mediator of an environment conducive to interventions directed at preventing bad nutrition (either due to excess or scarcity), and the role of the home in reinforcing a healthy food environment.

Accordingly, the objectives of this research were to compare the perceptions of parents, teachers, and experts of food environments at school that favored healthy habits in schoolchildren during the pre-Covid-19 stage; and also, to describe the perceptions of parents and teachers of food environments at home during the Covid-19 pandemic in Latin America.

## Material and methods

### Study design

This was an online, transversal study conducted in January 2021 using self-reporting among parents (PA), primary school teachers (TE) and experts (EXP) on school food environments in Latin America (LA). PA were defined as primary caregivers (parents or guardians) of children, those found in the first line of children’s interpersonal environment and who have primary influence in the construction of their home food environments. TE were identified as the primary school teachers that facilitate teaching-learning processes of subjects and content related to healthy habits, both in-person and virtually; they were further identified as important actors that contribute to the modeling of the food environment in the school and classroom, and its possible influence at home. Using the community and organizational aspects of the eco-social model, the EXP on food environments were identified as academics in various disciplines connected to the health sector and to public or private institutions linked to public policy, who are charged with designing and implementing health promotion interventions in school, family and community contexts [21].

### Instrument design

**Fig 1:**
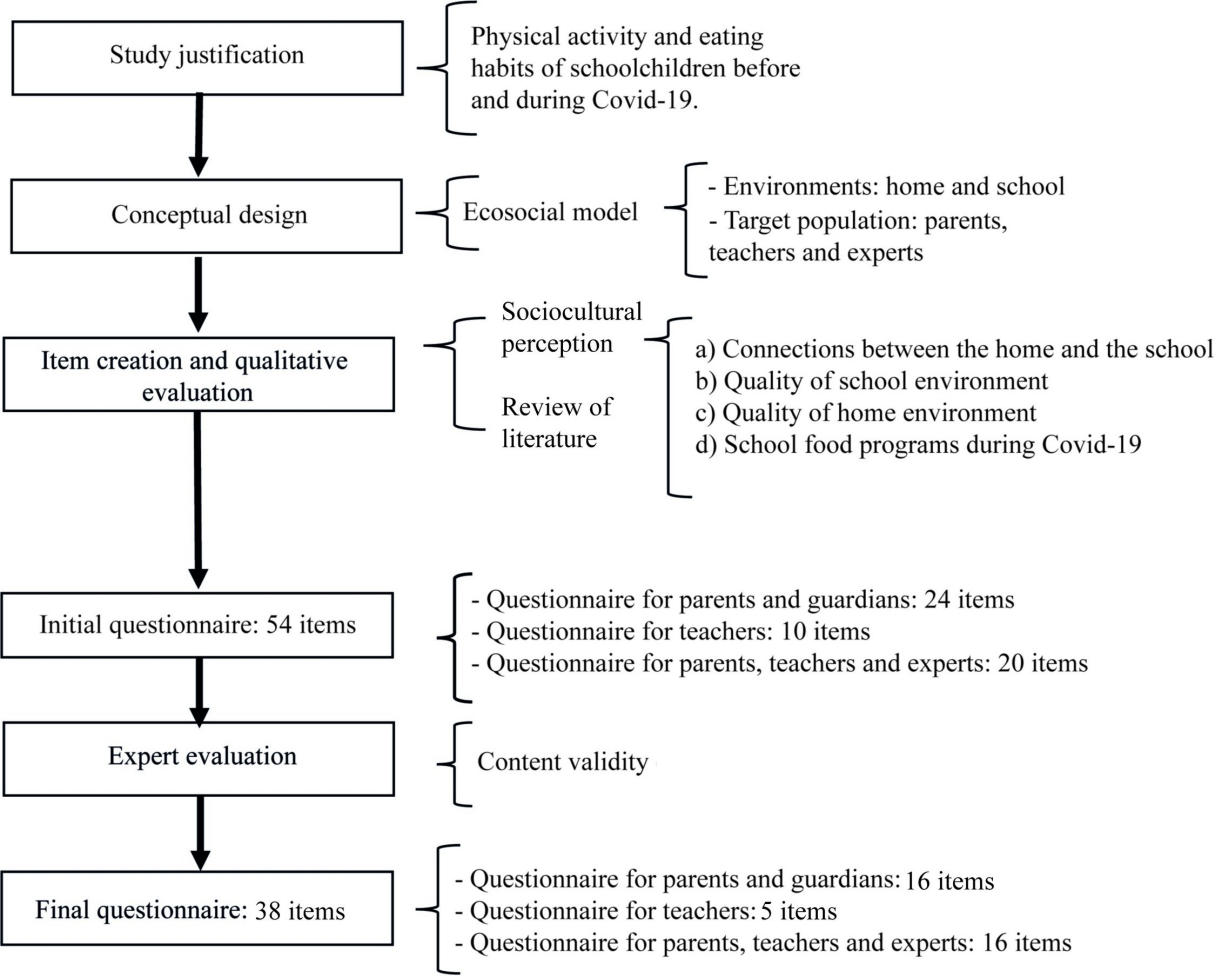
Design of the questionnaire perceptions of parents, experts and teachers in Latin America regarding the food environment in the home and connections with the school that promote healthy habits in students during the Covid-19 pandemic (3PyENSAN).

A questionnaire was designed and titled “Perceptions of parents, experts and teachers in Latin America regarding the food environment in the home and connections with the school that promote healthy habits in students during the Covid-19 pandemic” (abbreviated as 3PyENSAN from its initials in Spanish). The pre-Covid-19 definition of the sociocultural perception of the school food environment has been used, and the areas to be evaluated were defined using the eco-social model. They included food aid, broader food conditions, PhA, the lifestyles of children and their families, and the connections between the school and the home during Covid-19. Questionnaire objectives included identifying the perceptions of PA and TE of the immediate causes of children’s nutritional health and examining the perception of PA, TE and EXP on the level of importance of different elements of the school food environment. The items were validated by a group of experts (Fig 1).

Questionnaire items were submitted to a five-expert content validation process. The EXP evaluated the questionnaire clarity, internal coherence, use of adequate language, question objectives, and appropriate use of measurement scales (frequency and lLikert scale). For their application, the questions were adapted to a neutral language for various sociocultural contexts in Mexico (MX), Chile (CL) and other countries in LA.

The final version of the 3PyENSAN questionnaire consisted of 39 items: 16 Likert scale questions focused on the pre-Covid period, to identify the perception of PA, TE and EXP of the level of importance of various elements of school food environments promoting the development of healthy habits in children; 16 questions with Likert agreement-scale responses aimed at identifying the perceptions of PA of conditions at home and at school that favor their children’s healthy habits during the pandemic; and five Likert scale questions aimed at identifying the perceptions of TE of connections between the school and the home to promote the development of healthy habits in students (S1 Table). The internal consistency of the items per questionnaire was analyzed. Cronbach’s alpha was calculated and was 0.96 for the PA, TE and EXP perception questionnaire, 0.87 for the questions answered by TE and 0.92 for the questionnaire answered by PA. Regarding to exploratory factor analysis, the questionnaire for the perceived level of importance of PA, TE and EXP, the items were organized into 4 factors; for the questionnaire answered by PA and TE, the items were organized into two factors (S2 Table).

### Variables

The respondent profiles were regarded as independent variable, and the perceptions of these profiles were regarded as response variable.

#### Independent variables

Participants, when answering the questionnaire, identified themselves with basic demographic information: gender, country, and participant profile: PA, TE or EXP.

#### Dependent variable

Sociocultural perception was defined using the Vargas-Melgarejo perspective [22], as not only a cognitive process, but a process of classifying perceptions as a result of actors’ social, cultural, historical, and lived circumstances, in this case, the PA, TE and EXP actors. Thus these perceptions are the outcome of the order and the meaningfulness that these groups assign to school environments, understanding the school environment in a broad sense as both physical and social, internal and external to the subject and to the society [22].

### Study population, and recruitment of the sample

The population included adults of both sexes; an open invitation was made to a population of basic education TE, PA and EXP, residents of LA, who were interested in the topic and had access to the internet. A sample size was determined to compare two independent proportions, expecting a difference of 15% [23], with an alpha risk of 0.05, a beta risk of 0.2 (with a power of 80%) in a bilateral contrast, with a loss rate of 30%; requiring 138 subjects in each of the groups. The participants were recruited through social networks (Facebook) and e-mail, using a snowball technique and unsponsored social network announcements.

### Questionnaire application

The questionnaire was applied in the context of the “Building healthy lives at school and at home” International Seminar-Workshop (*Construyendo una vida saludable en la escuela y el hogar;* https://pesoeh.wixsite.com/pesoeh/seminario-taller), and as part of the formative research process of the “Development of a Model for Healthy, Sustainable School Environments” project (*Desarrollo de un Modelo de Ambientes Escolares Saludables Sustentables*). A person interested in attending the above-mentioned webinar received the questionnaire when they completed their registration; to raise the response rate, they then received a series of three emails inviting them to fill out the form. The response period was from January 16 to February 3, 2021, and the event was held on January 21 and 22; 80.3% of the responses were obtained before the event, from the following countries: Argentina, Chile, Colombia, Costa Rica, Dominican Republic, Ecuador, Guatemala, Honduras, Mexico, Nicaragua, Panama, Peru, Paraguay, El Salvador, Uruguay and Venezuela. Questionnaires were filled out with self-reporting online using Google Forms. An introduction and a description of the questionnaire objectives was included, as well as a privacy and confidentiality notice regarding the information obtained, (the choice to complete the questionnaire was optional). Identification data was requested from participants: email address, profile (PA, TE and EXP) and home country; 371 forms were eliminated from participants who responded more than once, and/or responded with different profiles than that according to the email address with which they registered for the event.

### Statistical analysis

Participant profile characteristics were described using proportions. The Likert scale had a maximum of five points for the answer “strongly agree” or “very important” and one point for the answer “strongly disagree” or “not important”. Due to the structure and nature of the data obtained, methods for categorical variables were used in their statistical treatment. A Fisher’s exact test was applied to determine the difference between the categories of responses of each actor; considering that the frequency of the answers rate was less than five in the category evaluated [24]. The analysis of association to determine the dependence between the responses of the actors or by country to the item of the questionnaires focused on the perception of the level of importance or level of agreement on the elements or actions that promote healthy food environments at school and at home, both in the pre-COVID 19 stage (for the general questionnaire), and for the questionnaires that referred to their items for the confinement stage (S1 Table). The data presented in a mosaic diagram as a graphic method to visualize data of the qualitative variables (response categories). In this graph, the independence is presented if the boxes across categories all have the same area [25]. When there was a statistically significant association in the bivariate analysis, an ordinal logistic regression model was run; and to measure the multivariate associations between the explanatory variables, the probability of the response categories per profile of the adjusted models was determined and plotted, with 95% confidence interval (CI) and respective P-value [26]. It was considered statistically significant when p<0.05; all analyses were performed using STATA 14 software for Mac.

### Ethical aspects

The study protocol was revised and accepted by the coordination of projects of the Executive Secretary of the Choose Healthy Living program (Elige Vivir Sano) of the Minister of Family and Social Development of the Government of Chile. At the time of opening the google forms file, before the questions section, the questionnaire displayed a text informing the participants about the use of the data for research purposes, the guarantee of anonymity and confidentiality, according to Google’s privacy policy (https://policies.google.com/privacy?hl=in); as well as the voluntary permanence in the participation of the survey. The participants acknowledged their consent to participate in electronic form through their register before answering the questionnaire. The information collected with the 3PyENSAN questionnaire does not confer sensitive data of the participants; and according to the declaration of Helsinki this research has considered low risk.

## Results

The study obtained N = 954 completed questionnaires, of which 48.4% were EXP (n = 462), 32.0% were TE (n = 305), and 19.6% were PA (n = 187). From the respondents of the PA questionnaire, 77% (n = 144) were Mexican, and the rest were from Chile and other LA countries (Table 1).

**Table 1:** Gender, country, and profile of participants.

		Female		Male		Total	
		n	%	n	%	n	%
		767	80.4	187	19.6	954	100
Actors							
	*Parents*	175	93.6	12	6.4	187	19.6
	*Teachers*	227	74.4	78	25.6	305	32.0
	*Experts*	365	79.0	97	21.0	462	48.4
Country							
	*Mexico*	618	81.2	143	18.8	761	79.8
	*Chile*	77	82.8	16	17.2	93	9.7
*Other countries LA* *		72	72.0	28	28.0	100	10.5

* Argentina, Colombia, Costa Rica, Dominican Republic, Ecuador, Guatemala, Honduras, Nicaragua, Panama, Peru, Paraguay, El Salvador, Uruguay, and Venezuela.

**Fig 2:**
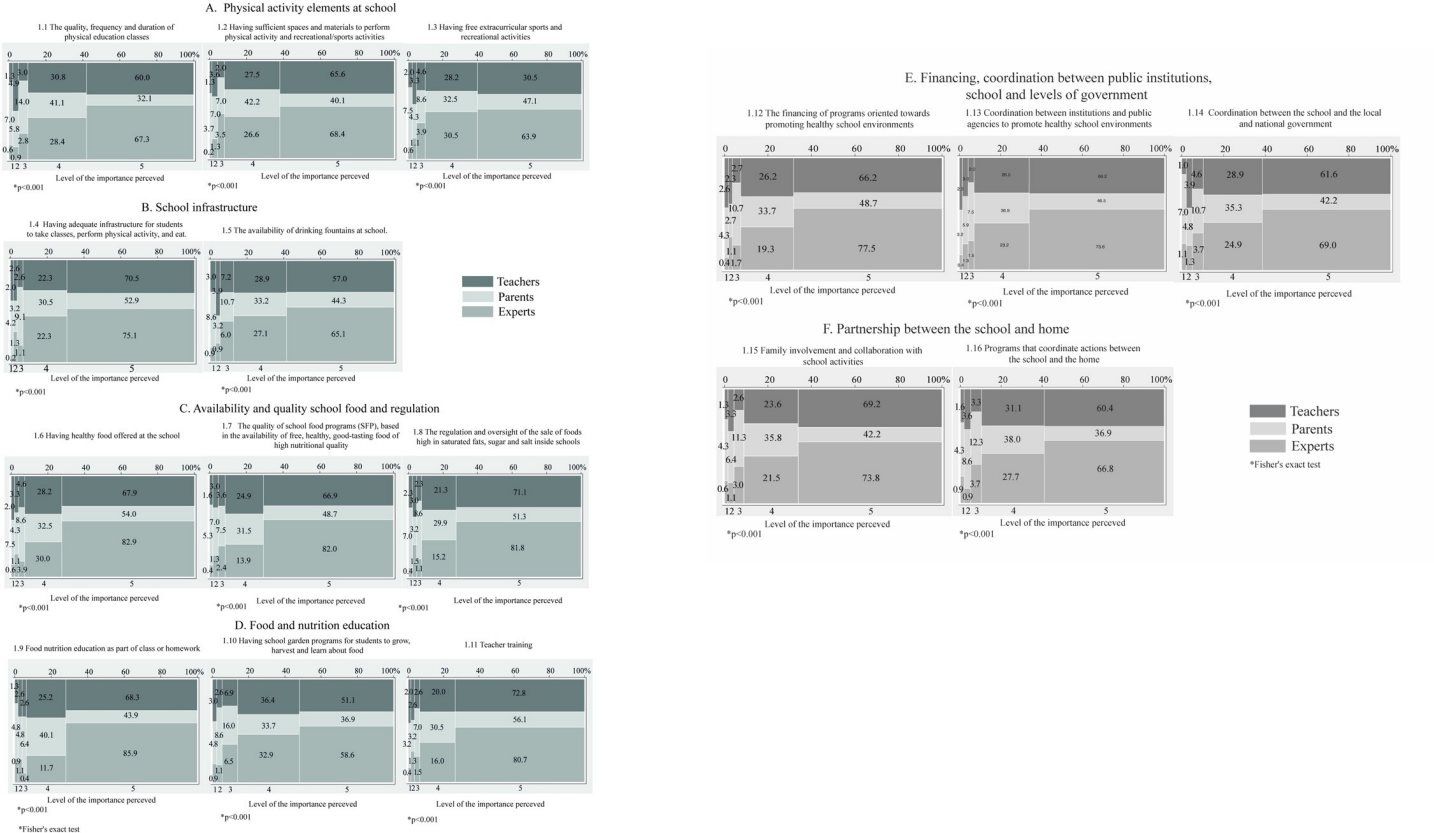
Mosaic plots about the level of importance perceived by parents (PA), teachers (TE) and experts (EXP) regarding the elements of creating healthy food environments at school, and promoting the development of healthy habits among children in Mexico, Chile, and other countries in Latin America (LA).

Concerning the objective to compare the perceptions between PA, TE, and EXP of food environments at school, there were statistically significant differences in all the aspects that were asked (<0.001). It was found that over 60% of TE and EXP believe that the quality, frequency and duration of physical education classes; spaces and materials to perform PhA and sports; and extracurricular sports and recreational activities are all very important (Fig 2A; item 1.1, 1.2, and 1.3). Over 70% of TE and EXP reported that having adequate infrastructure for students to hold classes, perform PhA, and eat is very important, but the proportion of PA with this same perception was lower (Fig 2B; item 1.4). A high proportion (65.1%) of EXP consider drinking fountains at school to be very important; they were followed by TE (57%), and then by a lower proportion of PA (44.3%) (Fig 2B; item 1.5). Regarding the availability and quality of school foods, SFP, and the regulation and sale of food at school, over 80% of EXP believed these to be very important, as did close to 70% of TE, and about 50% of PA also believed that (Fig 2C; items 1.6, 1.7 and 1.8). Most EXP (85.9%) identified the elements of the school environment related to food and nutrition education (FNE) within homework or classes as very important; this was followed by 68.3% of TE and a lower proportion of PA (43.9%). Over 50% of TE and EXP identified the existence of school garden programs for growing, harvesting and learning about food as very important, while only 36.9% of PA feel the same. High proportions of EXP (80.7%) and TE (72.8%) believe that teacher training is very important, while 56% of PA agreed with them (Fig 2D; item 1.9, 1.10, and 1.11). In the area of financing, coordination between public institutions, and coordination between the school and different levels of government as elements that contribute to better healthy school environments, over 60% of EXP and TE identified this as very important, while less than 50% of PA did think so (Fig 2E; items 1.12, 1.13 and 1.14). With respect to the integration, collaboration and coordination undertaken to connect families to school activities, also as elements that help build healthy school environments, over 60% of EXP and TE perceive this as very important, while only 43% of PA hold the same belief (Fig 2F; items 1.15 and 1.16).

Logistic regression analysis shows the probability that PA, ET or EX have assigned a level of importance regarding the aspects or elements that support food environments in schools. The ratio of the logistic regression ordinal models was statistically significant (p <0.001); however, data adjusted for gender and country variables had no statistical significance.

**Fig 3:**
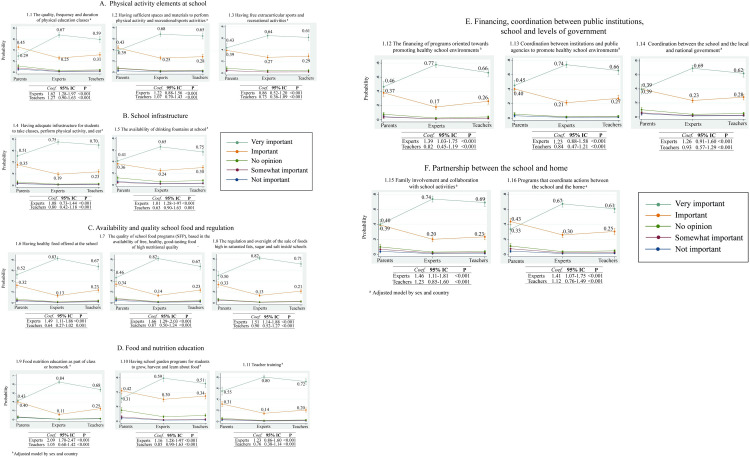
Probability regarding level of importance perceived by parents (PA), teachers (TE) and experts (EXP) regarding the elements of creating healthy food environments at school, and promoting the development of healthy habits among children in Mexico, Chile, and other countries in Latin America (LA).

For PhA items, the largest difference was shown in item 1.1; EXP were 38% more likely to perceive the quality, frequency and duration of physical education courses offered by the school as very important in compared to PA (67% of the EXP vs 29% of the PA); this aspect was less likely to be considered very important by PA (Fig 3A; item 1.1). Regarding the items at the school infrastructure, there was a difference of 24% between EXP and PA on the probability of perceiving it as very important to have an adequate infrastructure to have physical activity classes or a place to eat, as well as to have drinking fountains (Fig 3B). The differences between the probabilities of EXP perceived that it is very important to have healthy food at school, that SFP is free and of good quality, and that there is regulation of junk food at school vs PA, were 31% and up to 36% (Fig 3C; items 1.6,1.7 and 1.8). The probability that the EXP considered classes or homework about food nutrition education (FNE) to be very important was 84% vs. the PA, which was 43%, and 25% less probability to have the same level of importance as the TE vs. the PA (Fig 3D; item 1.9). Having indicators that the school has a garden and TE trained to teach about FNE is very important; the percentages go from 59% to 80% for EXP vs 31% and 51% for PA (Fig 3D; item 1.10 and 1.11). Having teachers trained to teach FNE got 55% probability by PA, the highest probability that PA considered very important (Fig 3D; item 1.11). Financing, coordination between public institutions, school and levels of government items the EXP were up to 77% probability to indicate these aspects as very important, while FA was up to 40% the likelihood to give the same level of importance (Fig 3E; items 1.12 and 1.13). Finally, in relation to the partnership of the school with the home, compared to the EXP, the PA were 34% less likely to consider the involvement and collaboration of the family in school activities, as well as programs to coordinate these actions. (Fig 3F; items 1.15 and 1.16).

**Fig 4:**
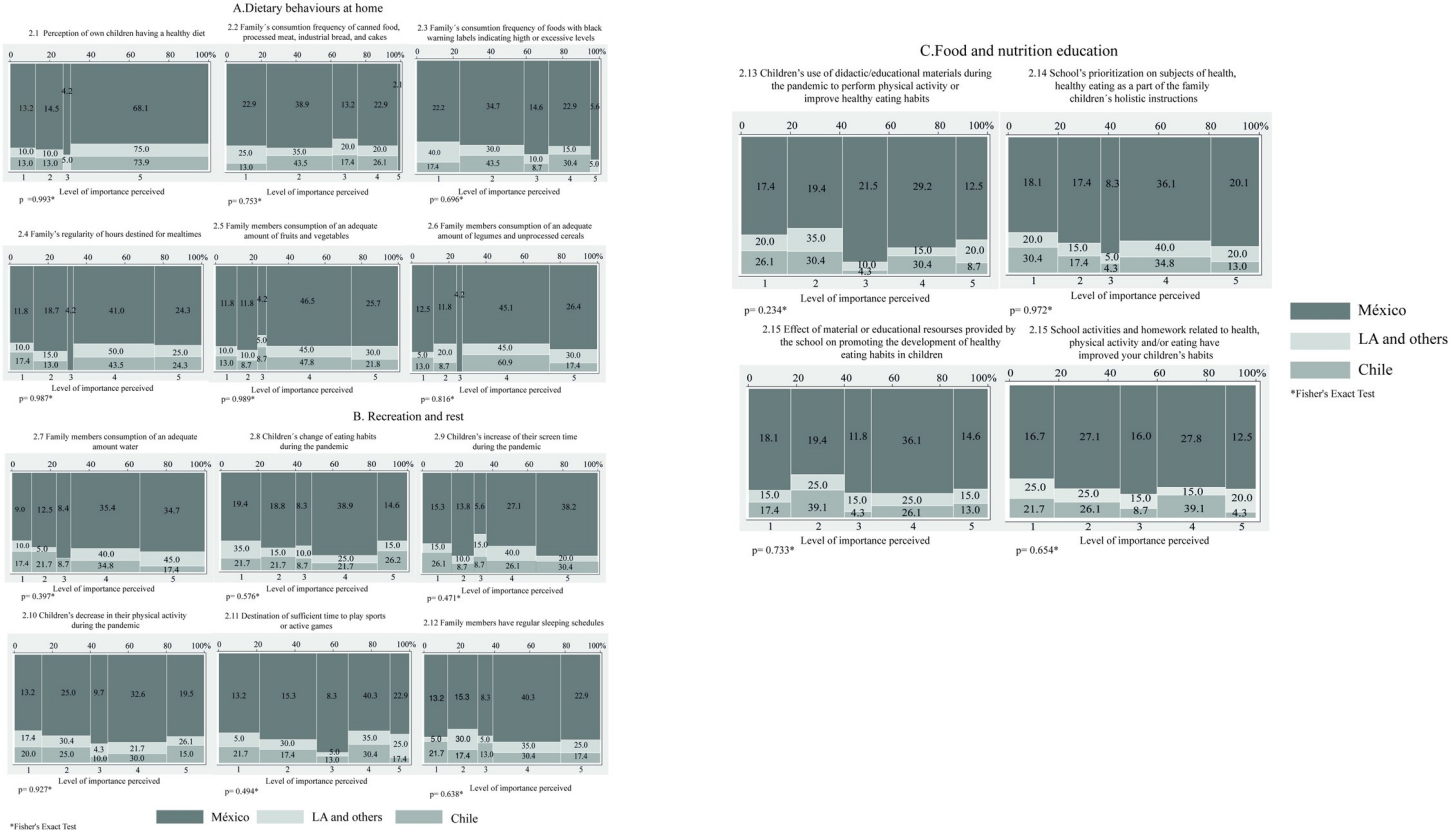
Mosaic plots about the perceptions of parents (PA) of conditions at home and at school that favor healthy habits in their children during the Covid-19 pandemic.

Regarding the perceptions of parent’s questionnaire of food environments at home during COVID-19 pandemic there was no statistical significant difference between Mexico, Chile and other LA countries (Fig 4). However, there were contrasting responses; PA reported either strongly agreeing over the quality of the environment and whether conditions at home and at school promoted healthy habits in their children during the pandemic, close to 70% of respondents expressed strong agreement that their children ate healthy food. (Fig 4A; item 2.1); approximately, between 61.8% and 56.5% of PA from Mexico, Chile and other countries from LA disagreed or strongly disagreed that members of their family frequently consumed canned food, processed meats, cakes or industrial bread (Fig 4A; item 2.2), or reported they disagreed or strongly disagreed that frequently consumed foods with one or more warning labels indicating excess sugar, fat or salt (Fig 4A; item 2.3). At the same time, over 67.8% of PA from Chile, 75% of PA from other countries in LA and 65.3% of PA from Mexico, responded strongly agreed and agreed that members of their family adhered to regular meal times, and approximately 70% agreed or strongly agreed that their family members consumed sufficient amounts of fruits and vegetables, legumes and unprocessed cereals, and drink water (Fig 4A; item 2.5, and 2.6). Also, more than 70% of PA from Mexico and other countries from LA perceived that their children adequately consume plain water (Fig 4A; item 2.7). The contrast were to the previous findings, half of PA agreed or strongly agreed that, during the pandemic, their children have changed their eating habits (Fig 3A; item 2.8) and have diminished their PhA (Fig 4; item 2.10); while 56.5% of the PA from Chile, and more than 60% of the PA from Mexico and other countries from LA, agreed or strongly agreed that their children have increased their screen time (Fig 4B; 2.9). One third of PA agreed or strongly agreed that they spend sufficient time in active games or activities with their children (Fig 4B; item 2.11). Additionally, 60% of PA agreed or strongly agreed that their family members have regular sleeping hours (Fig 4B; item 2.12). With respect to educational resources used during the pandemic, 40% of PA agreed or strongly agreed that their children had made use of educational and didactic materials on television or online to perform PhA and/or improve their eating habits (Fig 4C; item 2.13); and 55% of PA agreed or strongly agreed that the school prioritizes the subjects of health, healthy eating and PhA as part of their children’s holistic instruction (Fig 4C; item 2.14). In addition, 48% of PA reported agreeing or strongly agreeing that the didactic resources or materials provided by the school promote the development of healthy eating habits and PhA in their children (Fig 4C; item 2.15), while 40% stated that they agreed or strongly agreed that their children have improved their habits as a result of health-related school activities and homework (Fig 4C; item 2.16).

**Fig 5:**
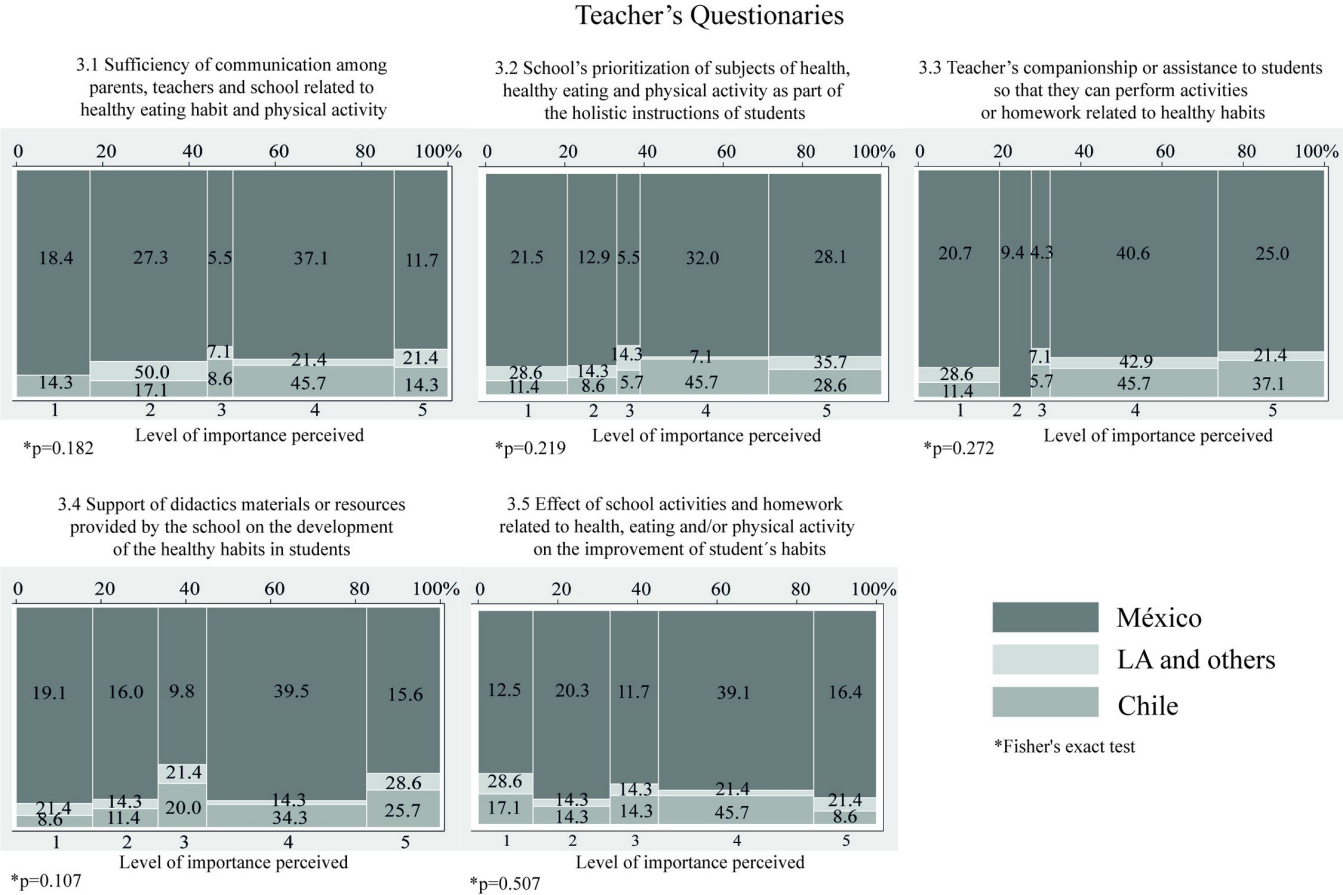
Mosaic plots about the perceptions of teachers (TE) of connections between the school and the home in order to promote the development of healthy habits among students during Covid-19.

On the subject of teacher’s perceptions of connections between the school and the home in order to promote the development of healthy habits among students during Covid-19, there were no statistically significant differences between countries. 48.8% of TE from Mexico, 42.8% of the TE from other LA countries, and 60% of the TE from Chile, stated that they agreed or strongly agreed that there is sufficient communication between the school, teachers and parents (Fig 5; item 3.1), and between 42.1% to 74.3% of TE reported agreeing or strongly agreeing that the school prioritizes the subjects of health, healthy eating and PhA as part of the integral instruction of students (Fig 5; item 3.2). 82.8% of the TE surveyed from Chile said that they agreed or strongly agreed that they had provided advice or accompaniment to students for activities and homework related to healthy eating habits and PhA, 17.2 and 18.5 percentage points less than TE from Mexico and other countries from LA, respectively (Fig 5; item 3.3); while 55% agreed or strongly agreed that school didactic materials and resources support the growth of students’ healthy eating habits and PhA (Fig 5; item 3.4), and 54.7% agreed or strongly agreed that students have improved their habits as a result of school activities and homework related to health, food and PhA (Fig 5; item 3.5).

## Discussion

Our findings showed that EXP, who are situated outside the interpersonal context of children, were more likely to consider very important the elements of the environment that contribute to develop healthy eating habits in schoolchildren, compared to PA, who are part of the interpersonal context of children. Therefore, our results could contribute to raise a reflection about the way in which many health and nutrition interventions have been designed, in other words, this reflection would help to question whether only the view of the EXP is sufficient to design interventions for the improvement of the food environment to prevent childhood OW/OB. On the other hand, the importance of having a mechanism in place to address the perceptions of PA in the design and evaluation of programs and public policies may be suggested. There is evidence of interventions based on the ecosocial model and action research, which showed the feasibility of incorporating the needs of the population from the diagnosis, design and implementation of actions to improve habits, carry out nutritional food education and physical activation actions. These interventions were more effective in the prevention of OW/OB, probably because they carried out actions that were more in line with the context [27,28].

In our study, the PA showed a low probability of considering very important the quality, frequency and duration of physical education classes. Which coincides with other studies, where these actors do not perceive physical education classes as one of the most relevant subjects in the formation of their children, giving greater importance to mathematics, languages, natural and social sciences [29]. As it is known, PhA is one of the main interventions aimed at promoting healthy lifestyles, and there is evidence of its benefits to improve executive functions and probably learning [30,31]. However, sustaining the benefits of these interventions remains a challenge if PhA is not valued in the same way as other subjects. Consequently, in some contexts it would be necessary to position PhA and active play within school as an important element for child learning and integral development [8].

In this research, the elements of infrastructure and materials for teaching classes, PhA and food consumption obtained a high probability of importance among TE and EXP, probably because school infrastructure has been identified as an internal environmental facilitator that influences the continued viability of interventions designed by EXP. In addition, TE consider that these elements are essential for teaching classes and PhA and having facilities that facilitate school coexistence and hygienic food consumption [32,33].

Before COVID-19, the availability, quality, regulation and monitoring of food in schools were the most common elements used to improve the school’s food environment. In our research findings, PA compared to EXP and TE were less probable to consider the availability and regulation of foods high in critical nutrients within the school as important. Some studies have suggested that PA tend to little support school laws and policies that improve and promote better nutrition of their children in schools, as well as rules prohibiting unhealthy foods [34,35].

The regulatory interventions are currently considered a priority by experts and researchers promoting specific public policy, such as taxes, regulation for the sale and advertising of food, and industrial food warning labelling, showing some degree in the reduction of consumption of packaged foods [36,37]. However, with the arrival of Covid-19 and confinement, it showed the vulnerability of the population and the increase of these products within households [38,39]. Accordingly, these results suggest the need to consider PA and their perceptions of these regulations. It has been reported that up to a third of PA may be neutral towards school feeding policies, they may not share an opinion or be against it [34].

At the same time, TE and PA showed similar probabilities with the level of importance of schools’ compliance with these rules [5]. Probably because in LA the sale of junk food within schools has been reported to generate resources for other school needs, which creates dissonance among the same members of the school community [40]. On the other hand, this does not prevent some PA from going against school feeding rules, arguing that they have little access to healthy food, as well as little time to prepare healthy meals [41]. The foregoing indicates that regulatory measures should consider social and psychological contexts, including parenting styles; that may present barriers to comply with these regulations. In addition, it has been identified that PA may have adapted to or normalized obesogenic environments to justify their own unhealthy behaviors [42,43].

In this research, it was considered that a relevant point was that few elements of the food environment obtained the same probability of being kept in mind by PA, TE and EXP with the same level of importance. In this case, it was FNE where there was a strong chance of considering this very important element. The EXP pointed out that the FNE should be part of the curriculum; and PA and TE considered the importance it should be given to the training of TE so that they can be facilitators of good eating habits and PA in their students. Some studies have identified teacher training as an indispensable element in promoting healthy habits in schools [44]. Conversely, the lack of teacher training has been identified as a barrier to maintaining health-promoting behavior interventions in schools [32].

In the elements of the FNE, having a school garden was less likely to be considered very important, both by the TE and by the EXP, and was the second element less likely to be considered very important by the PA. This finding could be relevant and considered for future designs of nutritional education interventions. In this regard it would be necessary to sensitize the school community on the importance of the school garden as a model for the development of educational skills and sustainability of the planet as well as to promote an increased consumption of vegetables and fruits [45,46]. Interventions with school gardens have shown some positive results, but limited, in increasing knowledge of health benefits and welfare aspects in schoolchildren, but being elements of the school environment that require a lot of PA participation, probably, that there is a major constraint to its prioritization [47,48].

On the other hand, the probability of considering as very important the elements related to financing, coordination between public institutions, school and government levels are very similar between TE and EXP. In this regard, it has been identified that investments in the education sector have great potential to improve nutritional outcomes and their long-term impact throughout the education of different generations. The fact that TE and EXP have similar perceptions in this area is probably due, as already mentioned, to the fact that TE are internal actors in the school context and can benefit from funding and good government relations through school feeding programs, improved sanitation and hygiene services, training in nutritional health education, among others [39,44]. Likewise, the EXP consider that actions within the educational sector are effective in the control and prevention of childhood OW/OB in LA countries [44].

Finally, in relation to the linking of the school with the home, the PA also showed a low probability of considering it as very important. This finding may seem contradictory of to the informed in literature; a qualitative review that interviews TE and school directors involved in programs related to healthy lifestyles showed that school staff wants the participation and support of the PA to help these interventions to succeed [44]. This conceptualization can be included in the educational model to stimulate the sensitivity in this issue between educational authorities, TE and students [49]. If these models propose the participation of PA to help model and teach healthy behaviors to students, formal support elements could be available so that in the future the responsibility of the family in their children’s school education could be assumed [35].

Regarding the perceptions of PA about the eating behaviors at home, the results were contrasting. On one side, they reported agreeing and strongly agreeing that household conditions promoted healthy habits in their children during the pandemic; family members were sticking to regular mealtimes, were consuming enough unprocessed fruits and vegetables, legumes and cereals; and drinking plain water; however, they were also agreeing or strongly agreeing that during the pandemic, their children changed their eating habits, decreased PA and increased screen time. The foregoing coincides with what was reported in other studies that were able to gather information about a pre- and post-confinement state. In this study, the context of households, as well as socioeconomic level and time to prepare food, had relation with having more family time and increased time to sleep, inasmuch as the time of transfer to schools or work has already suppressed. However, it has been reported at the same time, a decrease in active recreational PhA derived from the limited availability of space within the house or even linked to some confinement actions such as the closure of recreational spaces like parks or squares [50].

In this study, a high percentage of TE strongly agreed or agreed that their teaching practice contributes to fostering healthy habits in this context of confinement. In this regard, few reports have considered perceptions of teaching practice in a specific aspect of FNE. In this topic, qualitative results that are different than our findings were detected; in that study, TE have limited in carrying out FNE actions due to limited class time, physical distance from students, changes in food supply patterns, and even reduced attendance [51].

Part of the limitation of this study is the small number of participants from Chile and LA, which leads to a bias in the representativeness of the respondents according to the regions. In addition, it should have been considered that this study collected information from PA of school-age infants, mainly females. Another important limitation is the temporality of our results, as we were unable to measure the participant perceptions at various points throughout the pandemic. However, the data collected are within the dates of confinement and closure of schools when virtual or online classes have taught, so the questions were in line with the reality experienced by a large part of LA, especially Mexico, and where there was a promotion of resuming healthy habits as a strategy to prevent complications of COVID-19.

These results suggest the need for further research on stakeholder’s perceptions of the interpersonal environment of children, and those who are managers of such environments when making decisions in school food settings. At the time of this study the literature has studied several multicomponent interventions in the school context [31,32,44], these recognized the importance of the actors who are also considered relevant in this study. However, no studies or reports have explored the level of importance each stakeholder attaches to these components as a measure of working towards a common goal. There is limited literature that compiles the perceptions of actors in the school context regarding the wide range of elements that has been considered when designing food environment interventions. This provides rationale for considering the perception of PA in the design of food environment interventions to promote healthy habits in students [41,52].

## Conclusions

Our findings showed that PA had less probability to perceive as very important elements of the school food environment compared to EXP and TE. The three profiles were more likely to perceive the elements of food and nutrition education as very important; conversely, parents were less likely to give the same level of importance to the elements of regulation for healthy food at school. More research is required related to food environments to provide broader descriptions of the school context and explore the relevance of children’s interpersonal mediators for the design of interventions. In addition, during Covid-19 lockdown, the PA showed contradictory information about good eating habits at home; while TE considered adequate education about healthy lifestyles for students during the pandemic.

## Supporting information

S1 Table Design of questionnaire items that reveal the level of importance that parents, teachers and experts give to the elements of building healthy food environments at school, and promoting the development of healthy habits among children in Mexico, Chile and other Latin American countries. (PDF)

S2 Table Design of questionnaire items that explore parents’ perceptions of conditions in their home and their connections to the school related to the promotion of healthy habits in their children during the Covid-19 pandemic. (PDF)

S3 Table Design of questionnaire items exploring the perceptions of teachers regarding connections between the home and the school to promote the development of healthy habits in students during Covid-19. (PDF)

S4 Table Analysis of items to measure internal consistency alpha of the questionnaire for parents, teachers, and experts’ perceptions about the level of importance of elements to create healthy food environments at school and promote the development of healthy habits. (PDF)

S5 Table Factorial structure identified on the questionnaire for the level of importance perceived by parents (PA), teachers (TE) and experts (EXP) regarding the elements of creating healthy food environments at school, and promoting the development of healthy habits among children in Mexico, Chile, and other countries in Latin America (LA). (PDF)

S6 Table Analysis of items to measure internal consistency alpha of the questionnaire of conditions at home and at home and at school that favor healthy habits in their children during the Covid-19 pandemic. (PDF)

S7 Table Factorial structure identified on the parent’s questionnaire for conditions at home and at school that favor healthy habits in their children during the Covid- 19 pandemic. (PDF)

S8 Table Analysis of items to measure internal consistency alpha of the questionnaire for teachers’ perceptions of the connections between the school and the home in the promotion of the development of healthy habits in students during Covid-19. (PDF)

S9 Table Factorial structure identified on the questionnaire for teachers’ perceptions of the connections between the school and the home in the promotion of the development of healthy habits in students during Covid-19. (PDF)
