# Supplementary material for: Perceptions of food environments in the school and at home during Covid-19: An online cross-sectional study of parents, teachers and experts from Latin America
Source: PLoS One. 2023 Jun 29;18(6):e0287747. doi: 10.1371/journal.pone.0287747 (PMC10309603; doi:10.1371/journal.pone.0287747)
Supplement: S1 Table — (PDF) [file pone.0287747.s001.pdf]

S1 Table. Design of questionnaire items that reveal the level of importance that parents, teachers and experts give to the elements of building healthy food environments at school, and promoting the development of healthy habits among children in Mexico, Chile and other Latin American countries.				
QUESTION	ASPECT	INDICATOR	QUESTION OBJECTIVE	CONCEPTUAL FOUNDATION
1.1 How important is the quality, frequency and duration of physical education classes?	Quality of the school environment	Perception of the importance of the principal elements that constitute healthy food environments	To learn the perceptions of the importance given to the principal elements that contribute to creating healthy environments—specifically physical education classes—to promote the development of healthy habits in children as an indicator of the school environment quality.	There are structural and service elements on school grounds that are related to the promotion of healthy habits such as physical activity (1). Some schools may have an ideal structure for these activities, but the class designed for them may be of insufficient quality (2).
1.2 How important is it that there are sufficient spaces and materials to perform physical activity and recreational/sports activities?	Quality of the school environment	Perception of the importance of the school's structural elements	There are structural and service elements on school grounds that are related to the promotion of healthy habits such as physical activity. Some schools may have an ideal structure for these activities, and the school may be considered an ideal space to promote healthy lifestyles, and take actions to increase PhA and prevent infantile obesity. Owing to this, it was	There are structural and service elements on school grounds that are related to the promotion of healthy habits such as physical activity (1). Some schools may have an ideal structure for these activities, and the school may be considered an ideal space to promote healthy lifestyles, and take actions to increase PhA and prevent infantile obesity (3).

			relevant to collect the perceptions of the primary actors that contribute to the creation of food environments regarding the level of importance that they give to having adequate spaces and materials to perform physical activity and sports/recreational activities, as an indicator of the school environment quality.	
1.3 How important are free extracurricular sports and recreational activities?	Quality of the school environment	Perception of the importance of free extracurricular sports and recreational activities	The effectiveness of the use of physical activity to treat and prevent infantile obesity has been reported. Such interventions can be conducted outside of school hours. It was considered relevant to collect the perceptions of the principal actors that contribute to the creation of food environments regarding the existence of free extracurricular sports and recreational activities, as an indicator of the quality of the school environment.	The effectiveness of the use of physical activity to treat and prevent infantile obesity has been reported. Such interventions can be conducted outside of school hours (4).

1.4 How important is having adequate infrastructure for students to take classes, perform physical activity, and eat (dining hall or cafeteria)?	Quality of the school environment	Perception of the importance of the school's structural elements: spaces to perform physical activity and eat food	There are structural and service elements on school grounds that are related to the promotion of healthy habits such as physical activity. Some schools may have an ideal structure for these activities, thus it is important to learn about the perceptions of the principal actors that contribute to the creation of food environments regarding school infrastructure for performing physical activity and eating, as an indicator of the quality of the school environment.	There are structural and service elements on school grounds that are related to the promotion of healthy habits such as physical activity. Some schools may have an ideal structure for these activities (1, 2, 7).
1.5 How important is the availability of drinking fountains at school?	Quality of the school environment	Perception of the importance of the school's structural elements: drinking fountains	School infrastructure, such as the availability of drinking fountains, is relevant to the promotion or discouragement of healthy habits. Having drinking fountains in schools is related to the use and consumption of potable drinking water, and also decreasing the consumption of sugary drinks (whenever their consumption is	School infrastructure, such as the availability of drinking fountains, is relevant to the promotion or discouragement of healthy habits. Having drinking fountains in schools is related to the use and consumption of potable drinking water, and also decreasing the consumption of sugary drinks (whenever their consumption is limited in the

			limited in the school environment). Because of this, it is important to learn about the perceptions of the principal actors that contribute to the creation of food environments regarding the availability of drinking fountains at school, as an indicator of the quality of the school environment.	school environment) (1).
1.6 How important is it to have healthy food offered at the school?	Quality of the school environment	Perception of the importance of the type and availability of food at school	School policies are important to obtain quality food to be made available and distributed in school. Local reports have associated the oversight of establishments that supply schools with the high availability of foods considered unhealthy. Having healthy food offered at school is considered to be a factor that influences in the prevention of infantile obesity. Because of this, it is important to collect the perceptions of the principal actors that contribute to the creation of food	Schools are spaces that create food environments that can contribute to unhealthy habits, owing to the distribution of unhealthy food (8, 9). Latin American food environments often offer unhealthy foods. The existence of healthy food offered at schools is considered to be an influencing factor in the prevention of infantile obesity; conversely, the existence of unhealthy food offered can contribute to the increase of energy intake and infantile obesity (10).

			environments regarding the existence of healthy food in schools, as an indicator of the quality of the school environment.	
1.7 How important is the quality of school food programs, based in the availability of free, healthy, good-tasting food of high nutritional quality?	Quality of the school environment	Perception of the quality of school services: food program quality	Through programs that offer food, school grounds are spaces that can contribute to reducing food insecurity among the school population, above all in countries where the population has a high prevalence of food insecurity. These programs create part of the food environment in schools, which have been recognized for their capacity to be food education spaces, or spaces where unhealthy food is provided and there is a risk of overeating. Because of this, it was considered relevant to gather the perceptions of the principal actors that contribute to the creation of food environments regarding the level of importance they give to school food programs, as an indicator of the	Through programs that offer food, school grounds are spaces that can contribute to reducing food insecurity among the school population, above all in countries where the population has a high prevalence of food insecurity (11, 12). These programs create part of the food environment in schools, which have been recognized for their capacity to be food education spaces, or spaces where unhealthy food is provided and there is a risk of overeating (10, 13).

			quality of the school environment.	
1.8 How important is the regulation and oversight of the sale of foods high in saturated fats, sugar and salt inside schools?	Quality of the school environment	Perception of the importance of the type and availability of food at school	School policies are important to obtain quality food to be made available and distributed in school. Local reports have associated the oversight of establishments that supply schools with the high availability of foods considered unhealthy. Because of this, it important to collect the perceptions of the principal actors that contribute to the creation of food environments regarding the regulation and oversight of unhealthy foods inside schools, as an indicator of the quality of the school environment.	School policies are important to obtain quality food to be made available and distributed in school (2) Local reports have associated the oversight of establishments that supply schools with the high availability of foods considered unhealthy (10).
1.9 How important is having food nutrition education as part of class or homework?	Quality of the school environment	Perception of the importance of having nutritional education and content	Teaching health in schools and preventing chronic non-communicable diseases, in the curriculum or with specific interventions, have been recognized as non-physical elements that	Teaching health in schools and preventing chronic non-communicable diseases, in the curriculum or with specific interventions, have been recognized as non-physical elements that create the nutritional and food environment

			create the nutritional and food environment in schools. Nutritional health education has been shown to be an important element in the success of multicomponent interventions aimed at preventing infantile obesity, and it has been reported that these types of actions of well-received by the school community. Because of this, it is important to gather the perceptions of the principal actors that contribute to the creation of food environments regarding the importance of having nutritional education in the curriculum, as an indicator of the quality of the school environment.	in schools (7, 9, 14). Nutritional health education has been shown to be an important element in the success of multicomponent interventions aimed at preventing infantile obesity, and it has been reported that these types of actions of well-received by the school community (5).
1.10 How important are school garden programs for students to grow, harvest and learn about food?	Quality of the school environment	Perception of the importance of school garden programs	School gardens have been recognized as an educational tool that contributes to teachings related to healthy eating, openness to vegetables, and sustainability. They can be structural elements inside	School gardens have been recognized as an educational tool that contributes to teachings related to healthy eating, openness to vegetables, and sustainability. They can be structural elements inside school grounds that

			school grounds that contribute to the promotion of the consumption of fruits and vegetables. Because of this, it is important to learn about the perceptions of the principal actors that contribute to the creation of food environments regarding the importance of having school gardens, as an indicator of the quality of the school environment.	contribute to the promotion of the consumption of fruits and vegetables (15-17).
1.11 How important is teacher training?	Quality of the school environment	Perception of the importance of teacher training	Teacher training and the methodologies used to treat health-related subjects have been reported as a perceived need of teachers, as members of the community surrounding schoolchildren. This makes it important to learn the perceptions of the principal actors that contribute to the creation of food environments regarding teacher training, as an indicator of the quality of the school environment.	Teacher training and the methodologies used to treat health-related subjects have been reported as a perceived need of teachers, as members of the community surrounding schoolchildren (1, 5, 6).

1.12 How important is the financing of school programs oriented towards promoting healthy school environments?	Quality of the school environment	Perception of the importance of the presence and financing of programs	Sustainable, ongoing programs about healthy eating are part of the creation of healthy environments in schools. It is important to gather the perceptions of the principal actors that contribute to the creation of food environments regarding the financing of programs directed at promoting healthy school environments, as an indicator of the quality of the school environment.	Sustainable, ongoing programs about healthy eating are part of the creation of healthy environments in schools. The limited duration of some of these programs has been considered one of their weaknesses (5, 18).
1.13 How important is coordination between institutions and public agencies to promote healthy school environments?	Quality of the school environment	Perception of the importance of intersectorial coordination and collaboration	Qualitative studies have reported that institutional coordination is necessary to implement programs and content that promote healthy lifestyles, and a lack of coordination in implementing nutritional health programs may limit their effect. Because of this, it is important to examine the perceptions of the principal actors that contribute to the creation of food environments	Qualitative studies have reported that institutional coordination is necessary to implement programs and content that promote healthy lifestyles, and a lack of coordination in implementing nutritional health programs may limit their effect (8, 10, 19).

			regarding coordination between institutions and public agencies to promote healthy school environments, as an indicator of the quality of the school environment.	
1.14 How important is the coordination between the school and the local and national government?	Connections between the school and the home	Perception of the importance of intersectorial coordination and collaboration	Qualitative studies have reported that institutional coordination is necessary to implement programs and content that promote healthy lifestyles, and a lack of coordination in implementing nutritional health programs may limit their effect. Because of this, it is important to examine the perceptions of the principal actors that contribute to the creation of food environments regarding coordination between institutions and public agencies to promote healthy school environments, as an indicator of the quality of the school environment.	Intersectorial coordination is relevant in implementing programs and strategies to create healthy school environments (19, 20).

1.15 How important is family involvement and collaboration with school activities?	Connections between the school and the home	Perception of the importance of family collaboration in the context of connections between the school and the home	Interventions have been made that involve the principal actors that influence children's food education, both at home (parents) and at school (teachers). These collaborative actions have been reported to be beneficial to the prevention of infantile obesity. Likewise, a lack of coordination between parents and schools may limit the implementation of preventive programs. Because of this, it is important to learn about the perceptions of the principal actors that contribute to the creation of food environments regarding the coordination between institutions and public agencies to promote healthy school environments, as an indicator of the quality of the school environment.	Interventions have been made that involve the principal actors that influence children's food education, both at home (parents) and at school (teachers) (4, 21). These collaborative actions have been reported to be beneficial to the prevention of infantile obesity (22).

1.16 How important are programs that coordinate actions between school and the home?	Connections between the school and the home	Perception of the importance of intersectorial coordination and collaboration	Interventions have been made that involve the principal actors that influence children's food education, both at home (parents) and at school (teachers). These collaborative actions have been reported to be beneficial to the prevention of infantile obesity. Likewise, a lack of coordination between parents and schools may limit the implementation of preventive programs. Because of this, it is important to learn about the perceptions of the principal actors that contribute to the creation of food environments regarding the coordination between institutions and public agencies to promote healthy school environments, as an indicator of the quality of the school environment.	Interventions have been made that involve the principal actors that influence children's food education, both at home (parents) and at school (teachers) (4). These collaborative actions have been reported to be beneficial to the prevention of infantile obesity (21).

References

1. Harrison F, Jones AP. A framework for understanding school based physical environmental influences on childhood obesity. Health & Place. 2012;18(3):639-48.
2. Bassi S, Gupta VK, Park M, Nazar GP, Rawal T, Bhaumik S, et al. School policies, built environment and practices for non-communicable disease (NCD) prevention and control in schools of Delhi, India. PLOS ONE. 2019;14(4):e0215365.
3. Micha R, Karageorgou D, Bakogianni I, Trichia E, Whitsel LP, Story M, et al. Effectiveness of school food environment policies on children's dietary behaviors: A systematic review and meta-analysis. PLOS ONE. 2018;13(3):e0194555.
4. Siegrist M, Hanssen H, Lammel C, Haller B, Halle M. A cluster randomised school-based lifestyle intervention programme for the prevention of childhood obesity and related early cardiovascular disease (JuvenTUM 3). BMC Public Health. 2011;11(1):258.
5. Day RE, Sahota P, Christian MS. Effective implementation of primary school-based healthy lifestyle programmes: a qualitative study of views of school staff. BMC Public Health. 2019;19(1):1239.
6. Colonia García FD. Coordinación intersectorial en estrategias de escuelas saludables, factores inhibidores e impulsores en los casos de Norte de Santander y Risaralda [master Thesis]: Uniandes; 2016.
7. Bay JL, Hipkins R, Siddiqi K, Huque R, Dixon R, Shirley D, et al. School-based primary NCD risk reduction: education and public health perspectives. Health Promotion International. 2017;32(2):369-79.
8. Piaggio L, Concilio C, Rolón M, Macedra G, Dupraz S. Alimentación infantil en el ámbito escolar: entre patios, aulas y comedores. Salud Colectiva. 2011;7(2):199-213.
9. Harrison K, Bost KK, McBride BA, Donovan SM, Grigsby-Toussaint DS, Kim J, et al. Toward a Developmental Conceptualization of Contributors to Overweight and Obesity in Childhood: The Six-Cs Model. Child Development Perspectives. 2011;5(1):50-8.
10. Food and Agriculture Organization. El Ambiente Alimentario en las Escuelas, las Políticas de Alimentación Escolar y la Educación en Nutrición. S/F.
11. Aguirre Becerra H, García Trejo JF, Vázquez Hernández MC, Alvarado AM, Romero Zepeda H. Panorama general y programas de protección de seguridad alimentaria en México. Revista Médica Electrónica. 2017;39:741-9.
12. FAO. Documento interino de cuestiones sobre el Impacto del COVID-19 en la seguridad alimentaria y la nutrición (SAN). In: (GANESAN) GdANdEeSAyN, editor. Italia2020.
13. Sánchez-García R, Reyes-Morales H, González-Unzaga MA. Preferencias alimentarias y estado de nutrición en niños escolares de la Ciudad de México. Boletín Médico del Hospital Infantil de México. 2014;71(6):358-66.
14. Hrafnkelsson H, Magnusson KT, Thorsdottir I, Johannsson E, Sigurdsson EL. Result of school-based intervention on cardiovascular risk factors. Scandinavian Journal of Primary Health Care. 2014;32(4):149-55.
15. Castro DC, Samuels M, Harman AE. Growing healthy kids: a community garden-based obesity prevention program. Am J Prev Med. 2013;44(3 Suppl 3):S193-9.
16. Davis JN, Ventura EE, Cook LT, Gyllenhammer LE, Gatto NM. LA Sprouts: a gardening, nutrition, and cooking intervention for Latino youth improves diet and reduces obesity. J Am Diet Assoc. 2011;111(8):1224-30.
17. Gatto NM, Ventura EE, Cook LT, Gyllenhammer LE, Davis JN. LA Sprouts: a garden-based nutrition intervention pilot program influences motivation and preferences for fruits and vegetables in Latino youth. J Acad Nutr Diet. 2012;112(6):913-20.
18. Abdollahi M, Amini M, Kianfar H, Dadkhah Piraghag M, Eslami Amirabadi M, Zoghi T, et al. Qualitative study on nutritional knowledge of primary-school children and mothers in Tehran. 2008.
19. Talavera Ortega M, CatalánValentín G. Dificultades para el desarrollo de la educación para la salud en la escuela. Opiniones del profesorado. Didáctica de las ciencias experimentales y sociales. 2007;21:119-28.
20. Monsalve Lorente L. La educación para la salud en la escuela en la adquisicion de estilos de vida saludables. Revista Internacional de Educación y Aprendizaje. 2013;1(1):107-22.

21. Schaefer A, Winkel K, Finne E, Kolip P, Reinehr T. An effective lifestyle intervention in overweight children: One-year follow-up after the randomized controlled trial on "Obeldicks light". Clinical Nutrition. 2011;30(5):629-33.
22. Golley RK, Magarey AM, Daniels LA. Children's food and activity patterns following a six-month child weight management program. Int J Pediatr Obes. 2011;6(5-6):409-14.
23. Bruss MB, Morris J, Dannison L. Prevention of childhood obesity: Sociocultural and familial factors. Journal of the American Dietetic Association. 2003;103(8):1042-5.
24. Nazar G, Petermann-Rocha F, Martínez-Sanguinetti MA, Leiva AM, Labraña AM, Ramírez-Alarcón K, et al. Actitudes y prácticas parentales de alimentación infantil: Una revisión de la literatura. Revista chilena de nutrición. 2020;47:669-76.
25. Ammar A, Brach M, Trabelsi K, Chtourou H, Boukhris O, Masmoudi L, et al. Effects of COVID-19 Home Confinement on Eating Behaviour and Physical Activity: Results of the ECLB-COVID19 International Online Survey. Nutrients. 2020;12(6):1583.
26. Pietrobelli A, Pecoraro L, Ferruzzi A, Heo M, Faith M. Effects of COVID-19 Lockdown on Lifestyle Behaviors in Children with Obesity Living in Verona, Italy: A Longitudinal Study. 2020;28(8):1382-5.
27. Srour B, Fezeu LK, Kesse-Guyot E, Allès B, Méjean C, Andrianasolo RM, et al. Ultra-processed food intake and risk of cardiovascular disease: prospective cohort study (NutriNet-Santé). Bmj. 2019;365:l1451.
28. Rundle AG, Park Y, Herbstman JB, Kinsey EW, Wang YC. COVID-19–Related School Closings and Risk of Weight Gain Among Children. Obesity. 2020;28(6):1008-9.
29. Zachary Z, Brianna F, Brianna L, Garrett P, Jade W, Alyssa D, et al. Self-quarantine and weight gain related risk factors during the COVID-19 pandemic. Obesity research & clinical practice. 2020;14(3):210-6.
30. Sinisterra-Loaiza LI, Vázquez BI, Miranda JM, Cepeda A, Cardelle-Cobas A. Hábitos alimentarios en la población gallega durante el confinamiento por la COVID-19. Nutrición Hospitalaria. 2020;37:1190-6.
31. Olivares C S, Lera M L, Mardones H MA, Araneda F J, Olivares C MA, Colque M ME. Motivaciones y barreras para consumir 5 porciones de frutas y verduras al día en madres de escolares y profesores de enseñanza básica. Archivos Latinoamericanos de Nutrición. 2009;59:166-73.
32. McKee C, Long L, Southward LH, Walker B, McCown J. The Role of Parental Misperception of Child's Body Weight in Childhood Obesity. J Pediatr Nurs. 2016;31(2):196-203.
33. Baladia E, Martínez-Rodríguez R. Legumbres y salud: sumario de evidencias rápidas. RED-de Nutricioón Basada en Evidencias, 2016.
34. Aguirre-Loaiza H, Mejía-Bolaño A, Cualdrón J, Ospina S. Psychology, Physical Activity, and Post-pandemic Health: An Embodied Perspective. Frontiers in Psychology. 2021;12(406).
35. Pérez- Narváez MV, Tufiño A. Teleeducación y COVID-19. CienciAmérica. 2020;9(2):58-64.
36. Charro-Huerga E, Elena Charro M. Formación del profesor de primaria en educación para la salud. Didáctica de las ciencias experimentales y sociales. 2017;32(1):183-201.
37. Moreno-Murcia JA, Huéscar Hernández E, Nuñez Alonso JL, León J, Valero Valenzuela A, Conte L. Protocolo de estudio cuasi-experimental para promover un estilo interpersonal de apoyo a la autonomía en docentes de educación física. Cuadernos de Psicología del Deporte. 2019;19(2):83-101.
38. Montenegro S, Raya E, Navaridas F. Percepciones Docentes sobre los Efectos de la Brecha Digital en la Educación Básica durante el Covid -19. Revista Internacional de Educación para la Justicia Social. 2020;9(3):317-33.
