# Supplementary material for: Perceptions of food environments in the school and at home during Covid-19: An online cross-sectional study of parents, teachers and experts from Latin America
Source: PLoS One. 2023 Jun 29;18(6):e0287747. doi: 10.1371/journal.pone.0287747 (PMC10309603; doi:10.1371/journal.pone.0287747)
Supplement: S2 Table — (PDF) [file pone.0287747.s002.pdf]

S2 Table. Design of questionnaire items that explore parents' perceptions of conditions in their home and their connections to the school related to the promotion of healthy habits in their children during the Covid-19 pandemic.				
QUESTION	ASPECT	INDICATOR	QUESTION OBJECTIVE	CONCEPTUAL FOUNDATION
How do you perceive the conditions in your home and at school for the promotion of healthy habits in your children during the Covid-19 pandemic?				
2.1 Do you believe that your children have a healthy diet?	Quality of the environment in the home	Perception of the quality of children's diet	To identify the perceptions that primary caregivers have about the quality of their children's diet, from the perspectives of their own sociocultural context, including their knowledge of the subject, cultural practices, and the influence of media and possibly teachers and the school. Responses are valid according to respondents' own perspective. The answers to this question can be compared to others on the questionnaire related to food groups considered to be either protective or unhealthy. This can help reveal possible conceptualizations of "healthy eating" inside the home.	Diet is influenced by sociocultural factors that may include beliefs and attitudes which are perceived as normal in certain age groups or situations (23, 24), although such practices may have unhealthy connotations (3). During the Covid-19 pandemic, both the negative effects of eating habits and an increase in obesogenic behavior have been reported (25, 26).

2.2 Do you believe that members of your family frequently consume canned food, processed meat, industrial bread, and cakes?	Quality of the environment in the home	Perception of the quality of the family diet	To learn about parents' perceptions of unhealthy habits, using the frequency of consumption of ultra-processed foods, within the family context during the pandemic. This answer can be compared with the question about perceptions of "healthy eating."	A high consumption of ultra-processed foods is associated with elevated cardiovascular risk (27). During the pandemic, it has been reported that the consumption of ultra-processed foods has increased, which is recognized as an obesogenic behavior (26, 28).
2.3 Do you believe that members of your family frequently consume foods with black warning labels indicating high or excessive levels of sugar, fat or salt, such as potato chips, cookies or crackers, candy, sugary juice or drinks, soda, boxed juice, fast food, etc.?	Quality of the environment in the home	Perception of the quality of the family diet	To learn about parents' perceptions of unhealthy habits, using the frequency of consumption of ultra-processed foods, within the family context during the pandemic. This answer can be compared with the question about perceptions of "healthy eating," and with the previous question about the consumption of processed foods and meats, industrialized bread, etc.	An increase in the consumption of snack food during the Covid-19 pandemic has been reported (26). The high consumption of these foods is associated with causal factors of obesity at early ages (27). This practice is recognized as an obesogenic behavior.
2.4 Do you believe that your family members have regular hours for mealtimes (breakfast, lunch and dinner)?	Quality of the environment in the home	Perception of the quality of the family diet	Parents' perceptions of possible disruptions in family members' mealtimes during the pandemic is considered relevant, because these behaviors are associated with actions such as snacking and the	Irregular mealtime hours are associated with behaviors such as grazing and snacking, and with foods of low nutritional quality and high caloric density,

			consumption of unhealthy foods. This can help reveal parents’ perceptions of possible risk behaviors associated with the development of infantile obesity.	as well as excess energy intake among children (25). During the confinement, an interruption in eating habits and food and meal routines inside the home has been reported, along with an increase in snacking and grazing (29).
2.5 Do you believe that your family members consume an adequate amount of fruits and vegetables?	Quality of the environment in the home	Perception of the quality of the family diet	To learn about parents’ perceptions of their family’s consumption of foods considered healthy during the pandemic, while comparing this with respondents’ perceptions recorded in the question about what they consider to be “healthy food.” To do this, it was considered relevant to learn how parents perceive the consumption of healthy foods such as fruits and vegetables.	Some studies have reported that, during the pandemic, the consumption of foods like fruits and vegetables has stayed the same as before, or even increased (30). However, it has also been reported that the consumption of fruits and vegetables, considered to be healthy foods with a protective effect on health, has decreased in some populations, particularly in children (25, 26). Barriers to eating healthy foods have been identified within parents’ eating habits (31, 32).

2.6 Do you believe that your family members consume an adequate amount of legumes (beans, lentils, soy) and unprocessed cereals?	Quality of the environment in the home	Perception of the quality of the family diet	To learn about parents' perceptions of their family's consumption of healthy foods during the pandemic, and at the same time compare these responses to those of the question about what they consider to be "healthy food."	The consumption of legumes as part of the family diet is a positive indicator of diet quality (30, 33).
2.7 Do you believe that the members of your family drink an adequate amount of water?	Quality of the environment in the home	Perception of the quality of the family diet	To learn about parents' perceptions of their family's consumption of healthy foods during the pandemic, and at the same time compare these responses to those of the question about what they consider to be "healthy food."	Some studies have reported an increase in the consumption of unhealthy drinks during the pandemic (25, 26, 30).
2.8 Do you believe that your children have changed their eating habits during the pandemic?	Quality of the environment in the home	Perception of the quality of children's diet	To learn about parents' perceptions of possible changes in their children's eating habits during the confinement. This can help understand parents' perceptions of their children's possible positive health-related behaviors (for example, now their children eat breakfast) as well as negative behaviors (for example, now their children eat or graze more). This question is related to question 1.2.15 about the resources used in schools to promote healthy eating habits and physical activity.	An increase in snacking and eating outside mealtimes has been reported during the pandemic (25). An increase in the consumption of unhealthy foods and risk behaviors for overweight and obesity has also been reported (29).

2.9 Do you believe that your children have increased their screen time (TV, computer, cellular, etc.) during the pandemic?	Quality of the environment in the home	Perception of the increase in children's screen time during the pandemic	To learn about parents' perceptions of their children's screen time during the pandemic, which can increase sedentary behaviors considered unhealthy.	Prolonged times in front of the screen have been associated with the development of obesity at early ages. An increase in the use of electronic devices has been reported during the Covid-19 pandemic (25, 26, 29).
2.10 Do you believe that your children have decreased their physical activity during the pandemic?	Quality of the environment in the home	Perception of children's sedentary behavior during the pandemic	This question is related to questions 1.2.11, 1.2.10 and 1.2.8, which look at parents' perceptions of changes (worsening) in PhA habits. This is relevant, as a decrease in children's physical activity has been reported during the pandemic.	A decrease in children's physical activity has been reported during the Covid-19 pandemic (26, 29, 34). It is relevant to learn about parents' perceptions of their children's physical activity.
2.11 Do you believe that you spend sufficient time playing sports or active games with your children, such as playing ball, jumping rope, or riding bicycles?	Quality of the environment in the home	Perception of spending sufficient time aiding or accompanying children in activities that get them physically active	To learn about parents' perceptions of practicing and promoting PhA with their children.	A decrease in physical activity among children has been reported during the Covid-19 pandemic. It is considered relevant to learn about parents' perceptions of the time that they spend promoting their children's physical activity (11, 25).
2.12 Do you believe that your family members have regular sleeping hours?	Quality of the environment in the home	Perception of family sleeping habits	To learn about parents' perceptions of their family's sleeping hours as an element of the habits considered healthy	Having appropriate rest times is related to an optimal level of growth and wellbeing.

			for family wellbeing.	Alterations in sleeping hours at home have been reported during the pandemic (25, 26, 29).
2.13 Do you believe that your children have made use of didactic or educational materials online or on television during the pandemic to perform physical activity and/or improve healthy eating habits?	Quality of the environment in the home	Perception of the use of resources for having a healthy life at home	To learn about parents' perceptions of the use of these materials to explore whether they are used to perform physical activity and/or improve eating habits.	During the pandemic, a decrease in physical activity and an increase in obesogenic behavior has been reported in children (28). In addition, various institutions (governments, non-profit organizations and schools) have distributed digital materials that promote physical activity and better eating habits among children (35).
2.14 Do you believe that the school prioritizes the subjects of health, healthy eating and physical activity as part of your children's holistic instruction?	Connections between the school and the home	Parents' perception of the importance that schools give to educational health activities	During the pandemic, it has been considered relevant to learn about parents' perspectives regarding the prioritization that schools gives to the subjects of health, eating and physical activity. This perspective can help show parents' level of awareness of school content and activity.	It is known that in schools, the primary subjects are math, language and natural sciences, while healthy eating and physical education are rarely given importance (19).
2.15 Do you believe that the materials or educational resources that the school provides (books, pamphlets, videos or classes)	Connections between the school and the home	Perception of the utility of resources aimed at promoting the development of	To learn about parents' perceptions of the utility of resources offered by the school that promote the	Factors associated with inappropriate weight gain in children have been identified

promote the development of healthy eating habits and physical activity in your children?		healthy habits in children	development of healthy habits in children. The school can play an important role in preventing overweight and obesity through taking actions related to healthy eating and physical activity.	in the school environment. However, the role of the school in preventing overweight and obesity has also been recognized, through actions and interventions related to healthy eating and physical activity (8, 10).
2.16 Do you believe that school activities and homework related to health, physical activity and/or eating have improved your children's habits?	Connections between the school and the home	Perception of the utility of school activities aimed at promoting the development of healthy habits in children	This question is related to question 1.2.12 about the utility of resources used in schools to promote healthy eating habits and PhA, and parents' perceptions of changes in children's eating habits. The role of the school in preventing overweight and obesity has been recognized, through actions and interventions related healthy eating and physical activity; these classroom actions can change school food environments and help direct students' behavior. Thus it is considered relevant to learn about parents' perceptions of existing school resources that promote healthy lifestyles and during the pandemic, and if they can transcend	The role of the school in preventing overweight and obesity has been recognized, through actions and interventions in healthy eating and physical activity. (14, 28)

			the home environment and cause changes in habits.	
During the Covid-19 pandemic, how frequently have the products of the school food program at your child's school arrived at your house (food basket, lunch box, school breakfast, etc.)?	School food programs during Covid-19	Description of the frequency of the reception of school food program support during the pandemic	To learn how frequently children receive the benefits of school food programs.	Heightened conditions of food insecurity have been observed during the pandemic, due to the suspension of food support in specific environments such as schools (12, 28).
During the Covid-19 pandemic, how frequently have you received either direct support or food subsidies through a social protection program?	School food programs during Covid-19	Description of the frequency of the reception of food support during the pandemic	To learn how frequently parents receive support from food programs during the pandemic.	Heightened conditions of food insecurity have been observed during the pandemic, due to the suspension of food support in specific environments such as schools (12, 28).

References

1. Harrison F, Jones AP. A framework for understanding school based physical environmental influences on childhood obesity. Health & Place. 2012;18(3):639-48.
2. Bassi S, Gupta VK, Park M, Nazar GP, Rawal T, Bhaumik S, et al. School policies, built environment and practices for non-communicable disease (NCD) prevention and control in schools of Delhi, India. PLOS ONE. 2019;14(4):e0215365.
3. Micha R, Karageorgou D, Bakogianni I, Trichia E, Whitsel LP, Story M, et al. Effectiveness of school food environment policies on children's dietary behaviors: A systematic review and meta-analysis. PLOS ONE. 2018;13(3):e0194555.
4. Siegrist M, Hanssen H, Lammel C, Haller B, Halle M. A cluster randomised school-based lifestyle intervention programme for the prevention of childhood obesity and related early cardiovascular disease (JuvenTUM 3). BMC Public Health. 2011;11(1):258.
5. Day RE, Sahota P, Christian MS. Effective implementation of primary school-based healthy lifestyle programmes: a qualitative study of views of school staff. BMC Public Health. 2019;19(1):1239.
6. Colonia García FD. Coordinación intersectorial en estrategias de escuelas saludables, factores inhibidores e impulsores en los casos de Norte de Santander y Risaralda [master Thesis]: Uniandes; 2016.
7. Bay JL, Hipkins R, Siddiqi K, Huque R, Dixon R, Shirley D, et al. School-based primary NCD risk reduction: education and public health perspectives. Health Promotion International. 2017;32(2):369-79.
8. Piaggio L, Concilio C, Rolón M, Macedra G, Dupraz S. Alimentación infantil en el ámbito escolar: entre patios, aulas y comedores. Salud Colectiva. 2011;7(2):199-213.
9. Harrison K, Bost KK, McBride BA, Donovan SM, Grigsby-Toussaint DS, Kim J, et al. Toward a Developmental Conceptualization of Contributors to Overweight and Obesity in Childhood: The Six-Cs Model. Child Development Perspectives. 2011;5(1):50-8.
10. Food and Agriculture Organization. El Ambiente Alimentario en las Escuelas, las Políticas de Alimentación Escolar y la Educación en Nutrición. S/F.
11. Aguirre Becerra H, García Trejo JF, Vázquez Hernández MC, Alvarado AM, Romero Zepeda H. Panorama general y programas de protección de seguridad alimentaria en México. Revista Médica Electrónica. 2017;39:741-9.
12. FAO. Documento interino de cuestiones sobre el Impacto del COVID-19 en la seguridad alimentaria y la nutrición (SAN). In: (GANESAN) GdANdEeSAyN, editor. Italia2020.
13. Sánchez-García R, Reyes-Morales H, González-Unzaga MA. Preferencias alimentarias y estado de nutrición en niños escolares de la Ciudad de México. Boletín Médico del Hospital Infantil de México. 2014;71(6):358-66.
14. Hrafnkelsson H, Magnusson KT, Thorsdottir I, Johannsson E, Sigurdsson EL. Result of school-based intervention on cardiovascular risk factors. Scandinavian Journal of Primary Health Care. 2014;32(4):149-55.
15. Castro DC, Samuels M, Harman AE. Growing healthy kids: a community garden-based obesity prevention program. Am J Prev Med. 2013;44(3 Suppl 3):S193-9.
16. Davis JN, Ventura EE, Cook LT, Gyllenhammer LE, Gatto NM. LA Sprouts: a gardening, nutrition, and cooking intervention for Latino youth improves diet and reduces obesity. J Am Diet Assoc. 2011;111(8):1224-30.
17. Gatto NM, Ventura EE, Cook LT, Gyllenhammer LE, Davis JN. LA Sprouts: a garden-based nutrition intervention pilot program influences motivation and preferences for fruits and vegetables in Latino youth. J Acad Nutr Diet. 2012;112(6):913-20.
18. Abdollahi M, Amini M, Kianfar H, Dadkhah Piraghag M, Eslami Amirabadi M, Zoghi T, et al. Qualitative study on nutritional knowledge of primary-school children and mothers in Tehran. 2008.
19. Talavera Ortega M, CatalánValentín G. Dificultades para el desarrollo de la educación para la salud en la escuela. Opiniones del profesorado. Didáctica de las ciencias experimentales y sociales. 2007;21:119-28.
20. Monsalve Lorente L. La educación para la salud en la escuela en la adquisicion de estilos de vida saludables. Revista Internacional de Educación y Aprendizaje. 2013;1(1):107-22.

21. Schaefer A, Winkel K, Finne E, Kolip P, Reinehr T. An effective lifestyle intervention in overweight children: One-year follow-up after the randomized controlled trial on “Obeldicks light”. Clinical Nutrition. 2011;30(5):629-33.
22. Golley RK, Magarey AM, Daniels LA. Children's food and activity patterns following a six-month child weight management program. Int J Pediatr Obes. 2011;6(5-6):409-14.
23. Bruss MB, Morris J, Dannison L. Prevention of childhood obesity: Sociocultural and familial factors. Journal of the American Dietetic Association. 2003;103(8):1042-5.
24. Nazar G, Petermann-Rocha F, Martínez-Sanguinetti MA, Leiva AM, Labraña AM, Ramírez-Alarcón K, et al. Actitudes y prácticas parentales de alimentación infantil: Una revisión de la literatura. Revista chilena de nutrición. 2020;47:669-76.
25. Ammar A, Brach M, Trabelsi K, Chtourou H, Boukhris O, Masmoudi L, et al. Effects of COVID-19 Home Confinement on Eating Behaviour and Physical Activity: Results of the ECLB-COVID19 International Online Survey. Nutrients. 2020;12(6):1583.
26. Pietrobelli A, Pecoraro L, Ferruzzi A, Heo M, Faith M. Effects of COVID-19 Lockdown on Lifestyle Behaviors in Children with Obesity Living in Verona, Italy: A Longitudinal Study. 2020;28(8):1382-5.
27. Srour B, Fezeu LK, Kesse-Guyot E, Allès B, Méjean C, Andrianasolo RM, et al. Ultra-processed food intake and risk of cardiovascular disease: prospective cohort study (NutriNet-Santé). Bmj. 2019;365:l1451.
28. Rundle AG, Park Y, Herbstman JB, Kinsey EW, Wang YC. COVID-19–Related School Closings and Risk of Weight Gain Among Children. Obesity. 2020;28(6):1008-9.
29. Zachary Z, Brianna F, Brianna L, Garrett P, Jade W, Alyssa D, et al. Self-quarantine and weight gain related risk factors during the COVID-19 pandemic. Obesity research & clinical practice. 2020;14(3):210-6.
30. Sinisterra-Loaiza LI, Vázquez BI, Miranda JM, Cepeda A, Cardelle-Cobas A. Hábitos alimentarios en la población gallega durante el confinamiento por la COVID-19. Nutrición Hospitalaria. 2020;37:1190-6.
31. Olivares C S, Lera M L, Mardones H MA, Araneda F J, Olivares C MA, Colque M ME. Motivaciones y barreras para consumir 5 porciones de frutas y verduras al día en madres de escolares y profesores de enseñanza básica. Archivos Latinoamericanos de Nutrición. 2009;59:166-73.
32. McKee C, Long L, Southward LH, Walker B, McCown J. The Role of Parental Misperception of Child's Body Weight in Childhood Obesity. J Pediatr Nurs. 2016;31(2):196-203.
33. Baladia E, Martínez-Rodríguez R. Legumbres y salud: sumario de evidencias rápidas. RED-de Nutricioón Basada en Evidencias, 2016.
34. Aguirre-Loaiza H, Mejía-Bolaño A, Cualdrón J, Ospina S. Psychology, Physical Activity, and Post-pandemic Health: An Embodied Perspective. Frontiers in Psychology. 2021;12(406).
35. Pérez- Narváez MV, Tufiño A. Teleeducación y COVID-19. CienciAmérica. 2020;9(2):58-64.
36. Charro-Huerga E, Elena Charro M. Formación del profesor de primaria en educación para la salud. Didáctica de las ciencias experimentales y sociales. 2017;32(1):183-201.
37. Moreno-Murcia JA, Huéscar Hernández E, Nuñez Alonso JL, León J, Valero Valenzuela A, Conte L. Protocolo de estudio cuasi-experimental para promover un estilo interpersonal de apoyo a la autonomía en docentes de educación física. Cuadernos de Psicología del Deporte. 2019;19(2):83-101.
38. Montenegro S, Raya E, Navaridas F. Percepciones Docentes sobre los Efectos de la Brecha Digital en la Educación Básica durante el Covid -19. Revista Internacional de Educación para la Justicia Social. 2020;9(3):317-33.
