# Supplementary material for: Perceptions of food environments in the school and at home during Covid-19: An online cross-sectional study of parents, teachers and experts from Latin America
Source: PLoS One. 2023 Jun 29;18(6):e0287747. doi: 10.1371/journal.pone.0287747 (PMC10309603; doi:10.1371/journal.pone.0287747)
Supplement: S3 Table — (PDF) [file pone.0287747.s003.pdf]

S3 Table. Design of questionnaire items exploring the perceptions of teachers regarding connections between the home and the school to promote the development of healthy habits in students during Covid-19.				
QUESTION	ASPECT	INDICATOR	QUESTION OBJECTIVE	CONCEPTUAL FOUNDATION
3.1 I believe that there is sufficient communication between parents, teachers and the school related to healthy eating habits and physical activity	Connections between the home and the school	Perception of communication between the members of the school community aimed at improving healthy habits in children	To learn about the perceptions of teachers of the sufficiency of the communication between distinct members of the school community directed at promoting healthy habits in children.	It has been reported that teachers, in developing health education, perceive little communication and coordination with institutions, management, colleagues and parents. This is perceived as a difficulty and limitation for these actors within the school environment (5, 19).
3.2 I think that the school prioritizes the subjects of health, healthy eating and physical activity as part of the holistic instructions of students.	Quality of the school environment	Perception of the importance of health education in school	To learn about teachers' perceptions of the prioritization that the school gives nutritional health.	Schools provide tangible elements, such as infrastructure, and intangible elements, such as processes that facilitate teaching and learning. These intangible elements include the treatment of subjects related to nutritional health (4). To treat these subjects, schools may give varying levels of importance to nutritional health subjects (3, 5, 20).

3.3 Do you accompany or assist your students so that they can perform activities or homework related to healthy eating habits and physical activity.	Connections between the school and the home	Accompaniment in school activities related to nutritional health	To learn about teachers' accompaniment of their students in activities or assignments related to healthy eating habits and physical activity.	It has been shown that the school has a relevant role to play in preventing overweight and obesity, through actions and interventions related to healthy eating and physical activity. Various limitations in treating these subjects in the classroom have also been described, including a lack of support from related institutions. This lack of support can translate into a lack of clear school policies and/or existing or available resources for this purpose (5, 19, 36, 37).
3.4 I believe that the didactic materials or resources offered by the school such as books, pamphlets, videos or courses support the development of healthy eating habits and physical activity in students.	Quality of the school environment	Perception of the usefulness of available resources	To learn about teachers' perceptions of the school's role in preventing overweight and obesity through actions and interventions related to physical activity and healthy eating. This question is related to question 1.2.15, about the utility of school resources employed to promote healthy	It has been shown that the school has a relevant role to play in preventing overweight and obesity, through actions and interventions related to healthy eating and physical activity (5, 14). Various limitations in treating these subjects in the

			eating and physical activity, and teachers' perceptions of changes in their students' eating habits.	classroom have also been described, such as a lack of support from related institutions (5). This lack of support can translate into a lack of clear school policies and/or existing or available resources for this purpose (2, 3, 5, 19).
3.5 I believe that school activities and homework related to health, eating and/or physical activity have resulted in an improvement in students' habits.	Connections between the school and the home	Perception of changes in students' nutritional health habits as a result of school activities	To learn about teachers' perceptions of improvement of nutritional health habits as a result of related activities and homework.	Teaching health in schools is relevant to the prevention of chronic non-communicable diseases (3, 14). Multiple strategies and contexts for action exist to teach these subjects in school. Activities and/or homework related to the promotion of healthy lifestyles has an impact in students' eating behavior and food environment. During the Covid-19 pandemic, various strategies have been used to educate the population, although many schools in Latin

				America remain closed at the date of this writing, and homework and school activities are conducted by distance learning using digital and/or virtual means (35, 38). Countries like Mexico currently use content with materials and lessons related to nutritional health, which are now further emphasized for the relevance they have during the contingency.

References

1. Harrison F, Jones AP. A framework for understanding school based physical environmental influences on childhood obesity. Health & Place. 2012;18(3):639-48.
2. Bassi S, Gupta VK, Park M, Nazar GP, Rawal T, Bhaumik S, et al. School policies, built environment and practices for non-communicable disease (NCD) prevention and control in schools of Delhi, India. PLOS ONE. 2019;14(4):e0215365.
3. Micha R, Karageorgou D, Bakogianni I, Trichia E, Whitsel LP, Story M, et al. Effectiveness of school food environment policies on children's dietary behaviors: A systematic review and meta-analysis. PLOS ONE. 2018;13(3):e0194555.
4. Siegrist M, Hanssen H, Lammel C, Haller B, Halle M. A cluster randomised school-based lifestyle intervention programme for the prevention of childhood obesity and related early cardiovascular disease (JuvenTUM 3). BMC Public Health. 2011;11(1):258.
5. Day RE, Sahota P, Christian MS. Effective implementation of primary school-based healthy lifestyle programmes: a qualitative study of views of school staff. BMC Public Health. 2019;19(1):1239.
6. Colonia García FD. Coordinación intersectorial en estrategias de escuelas saludables, factores inhibidores e impulsores en los casos de Norte de Santander y Risaralda [master Thesis]: Uniandes; 2016.
7. Bay JL, Hipkins R, Siddiqi K, Huque R, Dixon R, Shirley D, et al. School-based primary NCD risk reduction: education and public health perspectives. Health Promotion International. 2017;32(2):369-79.

8. Piaggio L, Concilio C, Rolón M, Macedra G, Dupraz S. Alimentación infantil en el ámbito escolar: entre patios, aulas y comedores. Salud Colectiva. 2011;7(2):199-213.
9. Harrison K, Bost KK, McBride BA, Donovan SM, Grigsby-Toussaint DS, Kim J, et al. Toward a Developmental Conceptualization of Contributors to Overweight and Obesity in Childhood: The Six-Cs Model. Child Development Perspectives. 2011;5(1):50-8.
10. Food and Agriculture Organization. El Ambiente Alimentario en las Escuelas, las Políticas de Alimentación Escolar y la Educación en Nutrición. S/F.
11. Aguirre Becerra H, García Trejo JF, Vázquez Hernández MC, Alvarado AM, Romero Zepeda H. Panorama general y programas de protección de seguridad alimentaria en México. Revista Médica Electrónica. 2017;39:741-9.
12. FAO. Documento interino de cuestiones sobre el Impacto del COVID-19 en la seguridad alimentaria y la nutrición (SAN). In: (GANESAN) GdANdEeSAyN, editor. Italia2020.
13. Sánchez-García R, Reyes-Morales H, González-Unzaga MA. Preferencias alimentarias y estado de nutrición en niños escolares de la Ciudad de México. Boletín Médico del Hospital Infantil de México. 2014;71(6):358-66.
14. Hrafnkelsson H, Magnusson KT, Thorsdottir I, Johannsson E, Sigurdsson EL. Result of school-based intervention on cardiovascular risk factors. Scandinavian Journal of Primary Health Care. 2014;32(4):149-55.
15. Castro DC, Samuels M, Harman AE. Growing healthy kids: a community garden-based obesity prevention program. Am J Prev Med. 2013;44(3 Suppl 3):S193-9.
16. Davis JN, Ventura EE, Cook LT, Gyllenhammer LE, Gatto NM. LA Sprouts: a gardening, nutrition, and cooking intervention for Latino youth improves diet and reduces obesity. J Am Diet Assoc. 2011;111(8):1224-30.
17. Gatto NM, Ventura EE, Cook LT, Gyllenhammer LE, Davis JN. LA Sprouts: a garden-based nutrition intervention pilot program influences motivation and preferences for fruits and vegetables in Latino youth. J Acad Nutr Diet. 2012;112(6):913-20.
18. Abdollahi M, Amini M, Kianfar H, Dadkhah Piraghag M, Eslami Amirabadi M, Zoghi T, et al. Qualitative study on nutritional knowledge of primary-school children and mothers in Tehran. 2008.
19. Talavera Ortega M, CatalánValentín G. Dificultades para el desarrollo de la educación para la salud en la escuela. Opiniones del profesorado. Didáctica de las ciencias experimentales y sociales. 2007;21:119-28.
20. Monsalve Lorente L. La educación para la salud en la escuela en la adquisicion de estilos de vida saludables. Revista Internacional de Educación y Aprendizaje. 2013;1(1):107-22.
21. Schaefer A, Winkel K, Finne E, Kolip P, Reinehr T. An effective lifestyle intervention in overweight children: One-year follow-up after the randomized controlled trial on “Obeldicks light”. Clinical Nutrition. 2011;30(5):629-33.
22. Golley RK, Magarey AM, Daniels LA. Children's food and activity patterns following a six-month child weight management program. Int J Pediatr Obes. 2011;6(5-6):409-14.
23. Bruss MB, Morris J, Dannison L. Prevention of childhood obesity: Sociocultural and familial factors. Journal of the American Dietetic Association. 2003;103(8):1042-5.
24. Nazar G, Petermann-Rocha F, Martínez-Sanguinetti MA, Leiva AM, Labraña AM, Ramírez-Alarcón K, et al. Actitudes y prácticas parentales de alimentación infantil: Una revisión de la literatura. Revista chilena de nutrición. 2020;47:669-76.
25. Ammar A, Brach M, Trabelsi K, Chtourou H, Boukhris O, Masmoudi L, et al. Effects of COVID-19 Home Confinement on Eating Behaviour and Physical Activity: Results of the ECLB-COVID19 International Online Survey. Nutrients. 2020;12(6):1583.
26. Pietrobelli A, Pecoraro L, Ferruzzi A, Heo M, Faith M. Effects of COVID-19 Lockdown on Lifestyle Behaviors in Children with Obesity Living in Verona, Italy: A Longitudinal Study. 2020;28(8):1382-5.
27. Srour B, Fezeu LK, Kesse-Guyot E, Allès B, Méjean C, Andrianasolo RM, et al. Ultra-processed food intake and risk of cardiovascular disease: prospective cohort study (NutriNet-Santé). Bmj. 2019;365:l1451.
28. Rundle AG, Park Y, Herbstman JB, Kinsey EW, Wang YC. COVID-19–Related School Closings and Risk of Weight Gain Among Children. Obesity. 2020;28(6):1008-9.
29. Zachary Z, Brianna F, Brianna L, Garrett P, Jade W, Alyssa D, et al. Self-quarantine and weight gain related risk factors during the COVID-19 pandemic. Obesity research & clinical practice. 2020;14(3):210-6.

30. Sinisterra-Loaiza LI, Vázquez BI, Miranda JM, Cepeda A, Cardelle-Cobas A. Hábitos alimentarios en la población gallega durante el confinamiento por la COVID-19. Nutrición Hospitalaria. 2020;37:1190-6.
31. Olivares C S, Lera M L, Mardones H MA, Araneda F J, Olivares C MA, Colque M ME. Motivaciones y barreras para consumir 5 porciones de frutas y verduras al día en madres de escolares y profesores de enseñanza básica. Archivos Latinoamericanos de Nutrición. 2009;59:166-73.
32. McKee C, Long L, Southward LH, Walker B, McCown J. The Role of Parental Misperception of Child's Body Weight in Childhood Obesity. J Pediatr Nurs. 2016;31(2):196-203.
33. Baladia E, Martínez-Rodríguez R. Legumbres y salud: sumario de evidencias rápidas. RED-de Nutricioón Basada en Evidencias, 2016.
34. Aguirre-Loaiza H, Mejía-Bolaño A, Cualdrón J, Ospina S. Psychology, Physical Activity, and Post-pandemic Health: An Embodied Perspective. Frontiers in Psychology. 2021;12(406).
35. Pérez- Narváez MV, Tufiño A. Teleeducación y COVID-19. CienciAmérica. 2020;9(2):58-64.
36. Charro-Huerga E, Elena Charro M. Formación del profesor de primaria en educación para la salud. Didáctica de las ciencias experimentales y sociales. 2017;32(1):183-201.
37. Moreno-Murcia JA, Huéscar Hernández E, Nuñez Alonso JL, León J, Valero Valenzuela A, Conte L. Protocolo de estudio cuasi-experimental para promover un estilo interpersonal de apoyo a la autonomía en docentes de educación física. Cuadernos de Psicología del Deporte. 2019;19(2):83-101.
38. Montenegro S, Raya E, Navaridas F. Percepciones Docentes sobre los Efectos de la Brecha Digital en la Educación Básica durante el Covid -19. Revista Internacional de Educación para la Justicia Social. 2020;9(3):317-33.
