# Supplementary material for: Perceptions of food environments in the school and at home during Covid-19: An online cross-sectional study of parents, teachers and experts from Latin America
Source: PLoS One. 2023 Jun 29;18(6):e0287747. doi: 10.1371/journal.pone.0287747 (PMC10309603; doi:10.1371/journal.pone.0287747)
Supplement: S4 Table — (PDF) [file pone.0287747.s004.pdf]

S4 Table. Analysis of items to measure internal consistency alpha of the questionnaire for parents, teachers, and experts' perceptions about the level of importance of elementsto create healthy food environments at school and promote the development of healthy habits.[a].

Item	Question	Correlation coefficient between scores at the item	Internal consistenc yalpha
	**Physical activity elements at school**		
1.1	The quality, frequency, and duration of physical education classes	0.735	0.969
1.2	Having sufficient spaces and materials to perform physical activity and recreational/sports activities	0.785	0.968
1.3	Having free extracurricular sports and recreational activities	0.791	0.968
	**School's infrastructure**		
1.4	Having adequate infrastructure for students to take classes, perform physical activity, and eat (dining hall or cafeteria)	0.828	0.967
1.5	The availability of drinking fountains at school	0.751	0.969
	**Availability and quality school food and regulation**		
1.6	Having healthy food offered at the school	0.694	0.970
1.7	The quality of school food programs (SFP), based in the availability of free, healthy, good-tasting food of high nutritional quality	0.804	0.968
1.8	The regulation and oversight of the sale of foods high in saturated fats, sugar, and salt inside schools	0.795	0.968
	**Food and nutrition education**		
1.9	Food nutrition education as part of class or homework	0.857	0.967
1.10	Having school garden programs for students to grow, harvest and learn about food	0.844	0.967
1.11	Teacher training	0.842	0.967
	**Financing, coordination between public**		
1.12	The financing of programs oriented towards promoting healthy school environments	0.845	0.967
1.13	Coordination between institutions and public agencies to promote healthy school environments	0.862	0.967

1.14	Coordination between the school and the local and national government	0.800	0.968
	**Partnership between the school and home**		
1.15	Family involvement and collaboration with school activities	0.851	0.967
1.16	Programs that coordinate actions between the school and the home	0.826	0.967
	**Total**		0.9697

[a] **Response category** Very important (5), Important (4), No opinion (3), Somewhat important (2), Not important (0)
