# Supplementary material for: Perceptions of food environments in the school and at home during Covid-19: An online cross-sectional study of parents, teachers and experts from Latin America
Source: PLoS One. 2023 Jun 29;18(6):e0287747. doi: 10.1371/journal.pone.0287747 (PMC10309603; doi:10.1371/journal.pone.0287747)
Supplement: S5 Table — (PDF) [file pone.0287747.s005.pdf]

S5 Table. Factorial structure identified on the questionnaire for the level of importance perceived by parents (PA), teachers (TE) and experts (EXP) regarding the elements of creating healthy food environments at school, and promoting the development of healthy habits among children in Mexico, Chile, and other countries in Latin America (LA).

	Item	***Factor 1*** *Organization and regulation in the community for healthy school food environments*	***Factor 2*** *School organization for Food and Nutrition Education*	***Factor 3*** *Partnership between the school and home*	***Factor 4*** *Complementary actions to improve healthy school food environments*
1.7	The quality of school food programs (SFP), based in the availability of free, healthy, good-tasting food of high nutritional quality	0.5248			
1.14	Coordination between the school and the local and national government	0.4858			
1.11	Teacher training	0.6103			
1.16	Programs that coordinate actions between the school and the home	0.5491			

1.4	Having adequate infrastructure for students to take classes, perform physical activity, and eat (dining hall or cafeteria)	0.7325			
1.12	The financing of programs oriented towards promoting healthy school environments	0.7763			
1.8	The regulation and oversight of the sale of foods high in saturated fats, sugar and salt inside schools	0.7231			
1.13	Coordination between institutions and public agencies to promote healthy school environments	0.7561			
1.6	Having healthy food offered at the school	0.6885			
1.1	The quality, frequency and duration of physical education classes		0.6124		
1.2	Having sufficient spaces and materials to perform physical activity and recreational/sports activities		0.6365		
1.9	Food nutrition education as part of class or homework		0.534		
1.15	Family involvement and collaboration with school activities			0.5774	
1.10	Having school garden programs for students to grow, harvest and learn about food				0.4908
1.5	The availability of drinking fountains at school				0.5112

1.3	Having free extracurricular sports and recreational activities	0.5019

*In this exploratory factor analysis exercise, the factor loadings generated a factor with only one item, which is related to family or parental involvement in school activities. We suggested generate more research on this topic, which could become a more complex construct.
