# Supplementary material for: Perceptions of food environments in the school and at home during Covid-19: An online cross-sectional study of parents, teachers and experts from Latin America
Source: PLoS One. 2023 Jun 29;18(6):e0287747. doi: 10.1371/journal.pone.0287747 (PMC10309603; doi:10.1371/journal.pone.0287747)
Supplement: S7 Table — (PDF) [file pone.0287747.s007.pdf]

S7 Table. Factorial structure identified on the parent's questionnaire for conditions athome and at school that favor healthy habits in their children during the Covid-19 pandemic.

Item	Question	**Factor 1** *Habits and resources for healthy living*	**Factor 2** *UPF[c] Consumption*
2.1	Do you believe that your children have a healthy diet?	0.7717	
2.4	Do you believe that your family members have regular hours for mealtimes (breakfast, lunch and dinner)?	0.8057	
2.5	Do you believe that your family members consume an adequate amount of fruits and vegetables?	0.8948	
2.6	Do you believe that your family members consume an adequate amount of legumes (beans, lentils, soy) and unprocessed cereals?	0.8704	
2.7	Do you believe that your family members drink an adequate amount of water?	0.8213	
2.8	Do you believe that your children have changed their eating habits during the pandemic?	0.5711	
2.9	Do you believe that your children have increased their screen time (TV, computer, cellular, etc.) during the pandemic?	0.598	
2.10	Do you believe that your children have decreased their physical activity during the pandemic?	0.5227	
2.11	Do you believe that you spend sufficient time playing sports or active games with your children, such as playing ball, jumping rope, or riding bicycles?	0.5484	
2.12	Do you believe that your family members have regular sleeping hours?	0.7838	
2.13	Do you believe that your children have made use of didactic or educational materials online or on television during the pandemic, to perform physical activity and/or improve healthy eating habits?	0.6802	

2.14	Do you believe that the school prioritizes the subjects of health, healthy eating and physical activity as part of your children's holistic instruction?	0.7586	
2.15	Do you believe that the materials or educational resources that the school provides (books, pamphlets, videos or classes) promote the development of healthy eating habits and physical activity in your children?	0.7318	
2.16	Do you believe that school activities and homework related to health, physical activity and/or eating have improved your children's habits?	0.6821	
2.2	Do you believe that members of your family frequently consume canned food, processed meat, industrial bread, and cakes?		0.6640
2.3	Do you believe that members of your family frequently consume foods with black warning labels indicating high or excessive levels of sugar, fat or salt,such as potato chips, cookies or crackers, candy, sugary juice or drinks, soda, boxed juice, fast food, etc.?		0.6789

[c] Ultraprocessed foodstuffs
