# Supplementary material for: Perceptions of food environments in the school and at home during Covid-19: An online cross-sectional study of parents, teachers and experts from Latin America
Source: PLoS One. 2023 Jun 29;18(6):e0287747. doi: 10.1371/journal.pone.0287747 (PMC10309603; doi:10.1371/journal.pone.0287747)
Supplement: S8 Table — (PDF) [file pone.0287747.s008.pdf]

S8 Table. Analysis of items to measure internal consistency alpha of the questionnaire for teachers' perceptions of the connections between the school and the home in the promotion of the development of healthy habits in students during Covid-19. [b]

Item	Question	Response category	Internal consistency alpha
3.1	I believe that there is sufficient communication between parents, teachers and the school related to healthy eating habits and physical activity	0.7585	0.8429
3.2	I think that the school prioritizes the subjects of health, healthy eating and physical activity as part of the holistic instructions of students	0.8174	0.8269
3.3	I accompany or assist my students so that they can perform activities or homework related to healthy eating habits and physical activity.	0.8549	0.8164
3.4	I believe that didactic materials or resources offered by the school such as books, pamphlets, videos or courses support the development of healthy eating habits and physical activity in students	0.8474	0.8209
3.5	I believe that school activities and homeworkrelated to health, eating and/or physical activity has resulted in an improvement in students' habits	0.3193	0.9335
	**Total**		0.8793

[b] **Response category**: Strongly agree (5), Agree (4), Neither agree nor disagree (3), Disagree (2), Strongly disagree (1).
