# Supplementary material for: Perceptions of food environments in the school and at home during Covid-19: An online cross-sectional study of parents, teachers and experts from Latin America
Source: PLoS One. 2023 Jun 29;18(6):e0287747. doi: 10.1371/journal.pone.0287747 (PMC10309603; doi:10.1371/journal.pone.0287747)
Supplement: S9 Table — (PDF) [file pone.0287747.s009.pdf]

S9 Table. Factorial structure identified on the questionnaire for teachers' perceptions of the connections between the school and the home in the promotion of the development of healthy habits in students during Covid-19.

Item	Question	Factor 1 *Elements to implementation food and nutrition education*	Factor 2 *Partnership between the school and home*
3.1	I believe that there is sufficient communication between parents, teachers and the school related to healthy eating habits and physical activity	0.807	
3.2	I think that the school prioritizesthe subjects of health, healthy eating and physical activity as part of the holistic instructions of students	0.869	
3.3	I accompany or assist my students so that they can performactivities or homework related to healthy eating habits and physical activity.	0.9193	
3.4	I believe that didactic materials or resources offered by the schoolsuch as books, pamphlets, videos or courses support the development of healthy eating habits and physical activity in students	0.9033	
3.5	I believe that school activities and homework related to health, eating and/or physical activity has resulted in an improvement in students' habits		-0.295

*In this exploratory factor analysis exercise, the factor loadings generated a factor with only one item, which is related to family or parental involvement in school activities. We suggested generate more research on this topic, which could become a more complex construct.
